# miRNA-196b inhibits cell proliferation and induces apoptosis in HepG2 cells by targeting IGF2BP1

**DOI:** 10.1186/s12943-015-0349-6

**Published:** 2015-04-08

**Authors:** Magali Rebucci, Audrey Sermeus, Elodie Leonard, Edouard Delaive, Marc Dieu, Maude Fransolet, Thierry Arnould, Carine Michiels

**Affiliations:** Laboratory of Biochemistry and Cellular Biology (URBC), NARILIS, University of Namur, 61 rue de Bruxelles, 5000 Namur, Belgium

**Keywords:** Apoptosis, Chemoresistance, Hypoxia, IGF2BP1, miR-196b

## Abstract

**Background:**

Tumor hypoxia is one of the features of tumor microenvironment that contributes to chemoresistance. miRNAs have recently been shown to play important roles in tumorigenesis and drug resistance. Moreover, hypoxia also regulates the expression of a series of miRNAs. However, the interaction between chemoresistance, hypoxia and miRNAs has not been explored yet. The aim of this study is to understand the mechanisms activated/inhibited by miRNAs under hypoxia that induce resistance to chemotherapy-induced apoptosis.

**Methods:**

TaqMan low-density array was used to identify changes in miRNA expression when cells were exposed to etoposide under hypoxia or normoxia. The effects of miR-196b overexpression on apoptosis and cell proliferation were studied in HepG2 cells. miR-196b target mRNAs were identified by proteomic analysis, luciferase activity assay, RT-qPCR and western blot analysis.

**Results:**

Results showed that hypoxia down-regulated miR-196b expression that was induced by etoposide. miR-196b overexpression increased the etoposide-induced apoptosis and reversed the protection of cell death observed under hypoxia. By a proteomic approach combined with bioinformatics analyses, we identified IGF2BP1 as a potential target of miR-196b. Indeed, miR-196b overexpression decreased IGF2BP1 RNA expression and protein level. The IGF2BP1 down-regulation by either miR-196b or IGF2BP1 siRNA led to an increase in apoptosis and a decrease in cell viability and proliferation in normal culture conditions. However, IGF2BP1 silencing did not modify the chemoresistance induced by hypoxia, probably because it is not the only target of miR-196b involved in the regulation of apoptosis.

**Conclusions:**

In conclusion, for the first time, we identified IGF2BP1 as a direct and functional target of miR-196b and showed that miR-196b overexpression reverses the chemoresistance induced by hypoxia. These results emphasize that the chemoresistance induced by hypoxia is a complex mechanism.

**Electronic supplementary material:**

The online version of this article (doi:10.1186/s12943-015-0349-6) contains supplementary material, which is available to authorized users.

## Background

Resistance mechanisms remain a key challenge in the fight against cancer. Acquired drug resistance is of major clinical significance both for conventional and targeted therapies [1]. The first cause of therapeutic failure results from genetic alterations existing before treatment. Several researchers have evidenced the role of epigenetic changes [2,3] as well as the influence of the tumor microenvironment [4] on chemoresistance. One of the main characteristics of tumor microenvironment is the presence of hypoxic areas. Hypoxia is known to affect gene expression not only by regulating transcription [5] but also by epigenetic changes [6]. Furthermore, hypoxia and the adaptive responses of cancer cells play an important role in cancer progression [5]. Tumor microenvironment also strongly modulates the sensitivity of cancer cells to therapies. Previous results from our group showed that hypoxia protects tumor cells from apoptosis induced by several chemotherapeutic agents [7,8]. This protective effect has also been demonstrated by others, using other cell lines and other drugs [9,10] suggesting a general process.

microRNAs (miRNAs) are 21–25 nucleotides small non-coding RNAs. In mammals, mature miRNAs regulate gene expression by directly degrading mRNA or by suppressing protein translation through binding to the 3′ untranslated region (3′UTR) of targeted gene transcripts [11]. A single miRNA can target hundreds of mRNAs and a single mRNA can be influenced by multiple miRNAs [12,13]. Hence, getting a comprehensive understanding of the phenotypic effects of miRNAs at the level of the entire cell might be difficult.

Recent studies have evidenced correlation between the expression of several miRNAs in tumors and their predictive values. The expression of different miRNAs seems to correlate with anti-cancer treatment sensitivity: miR-30c, miR-130a and miR-335 expression is decreased in different chemoresistant cell lines while Let-7 g and miR-181b are associated to a response to anti-metabolic agents such as 5-fluorouracil (for review [2-4]). Moreover, several miRNAs directly influence the sensitivity of tumor cells to anticancer agents. As examples, miR-155 modulates breast cancer cell sensitivity to doxorubicin, paclitaxel and etoposide [5], miR-148a decreases paclitaxel resistance of prostate cancer cells [14], miR-125b confers paclitaxel resistance in breast cancer cells by decreasing Bcl-2 expression [15], and the let-7 family of miRNAs inhibits Bcl-xl expression and potentiates sorafenib-induced apoptosis in human hepatocarcinoma [16].

In addition to modulate apoptosis, hypoxia also regulates the expression of several miRNAs [17]. Among these hypoxia-regulated miRNAs, the HIF-responsive miR-210 appears as a ubiquitously expressed hypoxamir [18-20].

If links between hypoxia and chemoresistance, between hypoxia and changes in miRNA expression and between miRNAs and chemoresistance have been established, no study until now has tried to integrate all three. Previous results from our group showed that hypoxia protects HepG2 cells against etoposide-induced apoptosis [7,8]. In this context, we first analyzed the expression of 365 miRNAs in HepG2 cells. Among the miRNAs potentially involved in chemoresistance induced by hypoxia, we identified miR-196b. Hypoxia decreased the miR-196b expression that was induced by etoposide. In addition, miR-196b overexpression reversed the protection induced by hypoxia. Furthermore, we identified IGF2BP1 (insulin-like growth factor 2 RNA binding protein1) as a direct and functional target of miR-196b. However, IGF2BP1 silencing was not sufficient to reverse the chemoresistance induced by hypoxia.

## Results

### Identification of miRNAs differentially expressed in cells incubated under normoxia or hypoxia with or without etoposide

**Figure 1:**
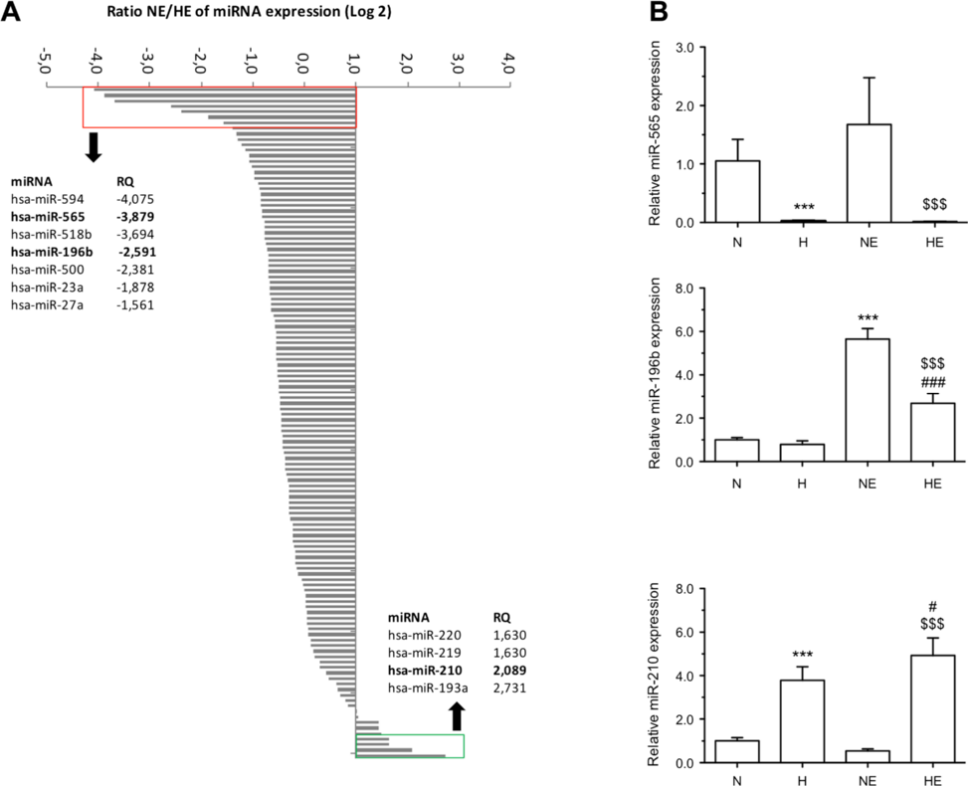
**miRNA expression profiling in HepG2 cells incubated under normoxia or hypoxia with or without etoposide.** Relative expression of 132 miRNAs was quantified by TLDA (TaqMan Low Density Array) in cells incubated under normoxia (N) or hypoxia (H) with or without etoposide (E) (50 μM) during 16 h. **(A)** Ratio of miRNA expression between cells incubated with etoposide under hypoxia in comparison to normoxia has been calculated. Four miRNAs were detected with an induction fold higher than 1.5 and seven miRNAs with a fold induction lower than - 1.5. Data were normalized to RNU44 and RNU48 endogenous controls using the threshold cycle method. **(B)** miRNA-565, −196b and −210 expression was monitored by conventional TaqMan RT-qPCR in HepG2 cells exposed to normoxia (N) or hypoxia (H) with or without etoposide (E) (50 μM) during 16 h. The relative expression of miRNA was determined using the threshold cycle method by normalizing to endogenous RNU44 (means ± 1SD, n = 3). ***: significantly different from normoxia (p < 0.001), #, ###: significantly different from hypoxia (p < 0.05, p < 0.001), $$$: significantly different from normoxia with etoposide (p < 0.001).

In order to explore the role of miRNAs in chemoresistance induced by hypoxia, the expression level of 365 miRNAs was compared in HepG2 cells exposed to normoxia or hypoxia with or without etoposide during 16 h. We identified 11 miRNAs for which expression was dysregulated with a fold change higher than 1.5 in cells incubated under normoxia with etoposide when compared to cells incubated under hypoxia with etoposide. The expression of 4 miRNAs was significantly up-regulated in cells incubated under hypoxia with etoposide compared to cells incubated under normoxia with etoposide (hsa-miR-220, −219, −210, −193a) while the expression of 7 miRNAs was significantly down-regulated in cells incubated under hypoxia compared to cells incubated under normoxia in the presence of etoposide (fold change > −1.5) (Figure 1A). To confirm the results of the TaqMan low-density array (TLDA) analysis, RT-qPCR assays were performed to evaluate miRNAs differentially expressed (Figure 1B). Among the 11 differentially expressed miRNAs identified by TLDA, changes in expression for only 3 miRNAs were validated. Etoposide significantly increased the miR-565 and miR-196b expression and hypoxia significantly decreased the expression of these two miRNAs induced by etoposide. On the contrary, hypoxia exposure with etoposide significantly increased miR-210 expression compared to cells incubated under normoxia with etoposide (Figure 1B). These results were consistent with the results obtained with the TLDA analysis.

However, miRBase.org indicated that the sequence of miR-565 is more likely a tRNA fragment and not a miRNA [21]. Thus, we decided to focus our study on the involvement of miR-210 and miR-196b in the chemoresistance induced by hypoxia.

### Anti-apoptotic effect of miR-210 overexpression and pro-apoptotic effect of miR-196b overexpression

**Figure 2:**
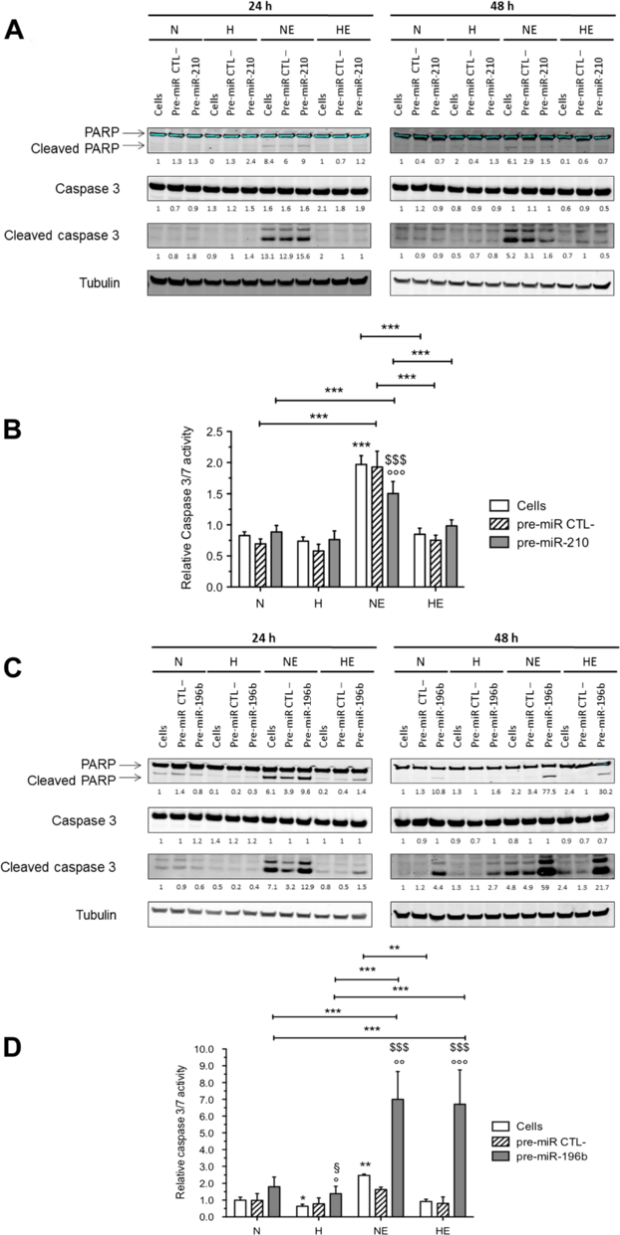
**Effect of miR-210 and miR-196b overexpression on the protective effect of hypoxia on the etoposide-induced apoptosis.** HepG2 cells were transfected with pre-miR negative control (pre-miR CTL-), pre-miR-210 or pre-miR-196b (50 nM) during 24 h or 48 h and then cells were incubated under normoxia (N) or hypoxia (H) with or without etoposide (E) (50 μM) during 16 h. **(A, C)** Cleaved caspase 3 and cleaved PARP were detected in total cell extracts by western blotting analysis, using specific antibodies. Immunodetection of α-tubulin was used to assess the total amount of proteins loaded on the gel. Numbers correspond to the quantification of the abundance of protein of interest normalized to the abundance of α-tubulin. **(B, D)** 48 h post-transfection, the caspase 3/7 activity was assayed by measuring free AFC released from the cleavage of the caspase 3/7 substrate Ac-DEVD-AFC. Results are expressed in fluorescence intensity, as means ± 1 SD (n = 3). *, **, ***: (p < 0.05, p < 0.01, p < 0.001), °, °°, °°°: significantly different from untransfected cells (Cells) (p < 0.05, p < 0.01, p < 0.001), $$$: significantly different from pre-miR negative control transfected cells (p < 0.001) respectively for each group (N, H, NE, HE).

We previously evidenced that hypoxia protects HepG2 cells from etoposide-induced apoptosis. In order to investigate the potential role of miR-196b and miR-210 in the hypoxia-induced resistance to etoposide, we performed western blot analysis for apoptotic markers in cells in which miR-210 or miR-196b was overexpressed by transfection with miRNA precursor (pre-miR). As shown in Figure 2, hypoxia decreased PARP and caspase 3 cleavage induced by etoposide both after 24 and 48 h incubation in the presence of etoposide in untransfected cells.

miR-210 overexpression decreased PARP and caspase 3 cleavage in cells exposed to normoxia with etoposide for 48 h, reaching level similar than the one observed in cells exposed to hypoxia with etoposide, in which miR-210 expression level is high (Figure 2A). These results were validated by a caspase 3/7 activity assay. Results showed that miR-210 overexpression significantly decreased the caspase 3/7 activity in cells incubated under normoxia with etoposide (Figure 2B). It must be noted that overexpression of miR-210 by pre-miR-210 transfection did not have any effect under hypoxia. Indeed, miR-210 is overexpressed under hypoxia and there is a strong protective effect of hypoxia on the etoposide-induced apoptosis. There is no difference in caspase 3 activity level, nor in caspase 3 and PARP cleavage in cells incubated with etoposide under hypoxia in comparison to control normoxic cells, so it is very unlikely that increasing miR-210 level could exert its anti-apoptotic function in these conditions. As our goal was to identify a miRNA that exerts a modulatory effect of the etoposide-induced apoptosis under hypoxia, we next focused our interest on the role of miR-196.

miR-196b overexpression strongly increased PARP and caspase 3 cleavage in cells incubated with etoposide under normoxia or hypoxia for 24 or 48 h (Figure 2C). As shown in Figure 2D, miR-196b overexpression induced a significant increase in caspase 3/7 activity. This activity reached comparable levels in cells incubated with etoposide under normoxia or hypoxia. Thus, miR-196b overexpression not only enhanced the apoptosis induced by etoposide under normoxia but also reversed the hypoxia-induced chemoresistance.

To further confirm the effect of miR-196b in hypoxia-induced chemoresistance, HepG2 cells were transfected with LNA-miR-196b (Locked Nucleic Acid) to inhibit the miR-196b expression. However, by using several LNA negative controls (LNA CTL- #1, LNA CTL- #2 or a mix of LNA CTL- #1 + #2), we observed that LNA negative controls had a strong impact on apoptotic markers studied (data not shown). It was thus impossible to interpret the results obtained with LNA-miR-196b.

Taken together, these results showed that miR-196b displays a pro-apoptotic function.

### Identification of miR-196b targets by proteomic analysis

**Figure 3:**
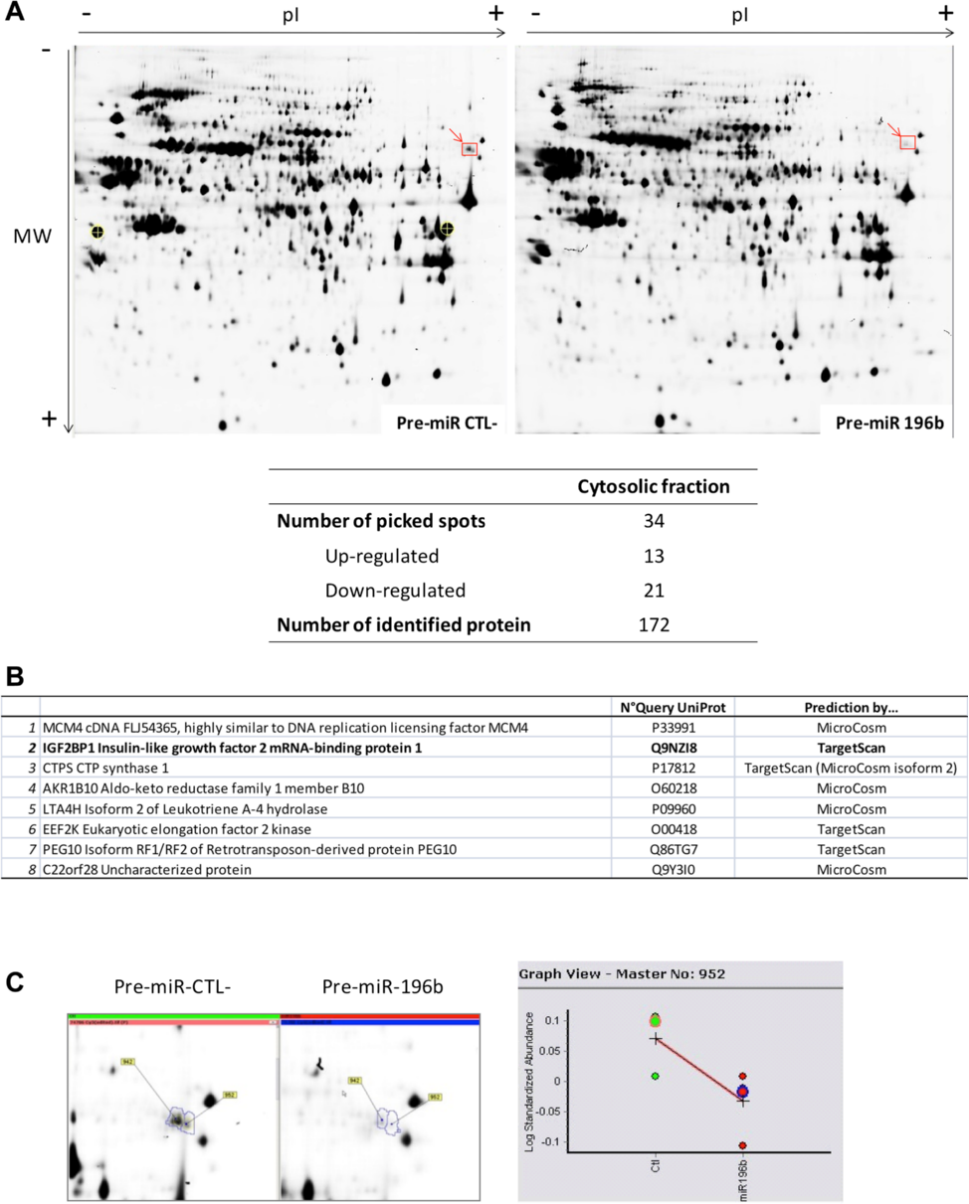
**Proteomic analysis after miR-196b overexpression in HepG2 cells.** HepG2 were transfected with pre-miR negative control or pre-miR-196b (50 nM) during 24 h and 2D-DIGE analysis of cytosolic fraction was performed (n = 4). **(A)** Proteins were loaded on IPG strips (18 cm, 3–11 non linear), followed by the second dimension on a vertical continuous 12.5% gel. Significantly down- or up-regulated proteins (cut-off 1.2, p value < 0.05) were identified and labeled with their serial number. Arrow: spot 952/942, IGF2BP1 protein. Table shows the number of down-regulated or up-regulated spots. **(B)** List of proteins identified by MS/MS analysis and predicted by softwares: TargetScan, MicroCosm and miRDB. **(C)** Intensity of spot 952 was significantly lower when HepG2 cells were transfected with pre-miR-196b versus pre-miR negative control during 24 h. Dot plots showed the variation between pre-miR negative control and pre-miR-196b for 4 replicates.

To unravel the potential role of miR-196b in HepG2 cells and to identify targets of miR-196b, we used a proteomic approach. HepG2 cells were transfected with pre-miR-196b or pre-miR negative control for 24 h. Cells were then harvested, lysed and subjected to subcellular fractionation to recover the cytosolic fraction. Relative protein abundance differences between negative control cells and cells that overexpress pre-miR-196b were then analyzed by 2D-DIGE gels in four independent replicates (Figure 3). Figure 3A shows a pair of representative 2-DE gel images of HepG2 cells transfected to pre-miR negative control compared to pre-miR-196b. Proteins from spots with significant changes in intensities (p < 0.05 up- or down-regulated) were cut out for identification by mass spectrometry. Thirty four spots differentially abundant were detected, of which 13 spots were up-regulated and 21 spots were down-regulated. The complete list of the 172 proteins identified by mass spectrometry is detailed in Additional file 1. We next compared the list of differentially expressed proteins from our proteomic study to the respective list of computationally predicted targets by several softwares (TargetScan, MicroCosm and miRDB) (Figure 3B). Since the number of differentially expressed proteins is typically much lower than the total number of predicted targets, we looked for proteins detected in our proteomic experiment that were also predicted as targets by the bioinformatics studies. We identified 8 differentially expressed proteins that were also predicted by at least one of the 3 algorithms as targets of miR-196b (Figure 3B). Among the 8 potential targets for miR-196b, three of them were involved in DNA (MCM4 cDNA and CTPS) or in protein (EEF2K) synthesis and the link between these processes and apoptosis is not clear. Similarly, AKR1B10 is an aldo-keto reductase enzyme that acts as all-trans-retinaldehyde reductase while LTA4H is an isoform of leukotriene A4 hydrolase. These metabolic pathways are most likely not involved either in regulating apoptosis. The last two proteins (retrotransposon-derived protein PEG10 and C22orf28) are of unknown function. We thus chose IGF2BP1 to investigate its role in the hypoxia-induced chemoresistance. IGF2BP1 was down-regulated by miR-196b overexpression as revealed by the zoom of spot 952 (Figure 3C) in the proteomic analysis as well as predicted to be a target of miR-196b by the TargetScan software.

### IGF2BP1 is a direct target of miR-196b

**Figure 4:**
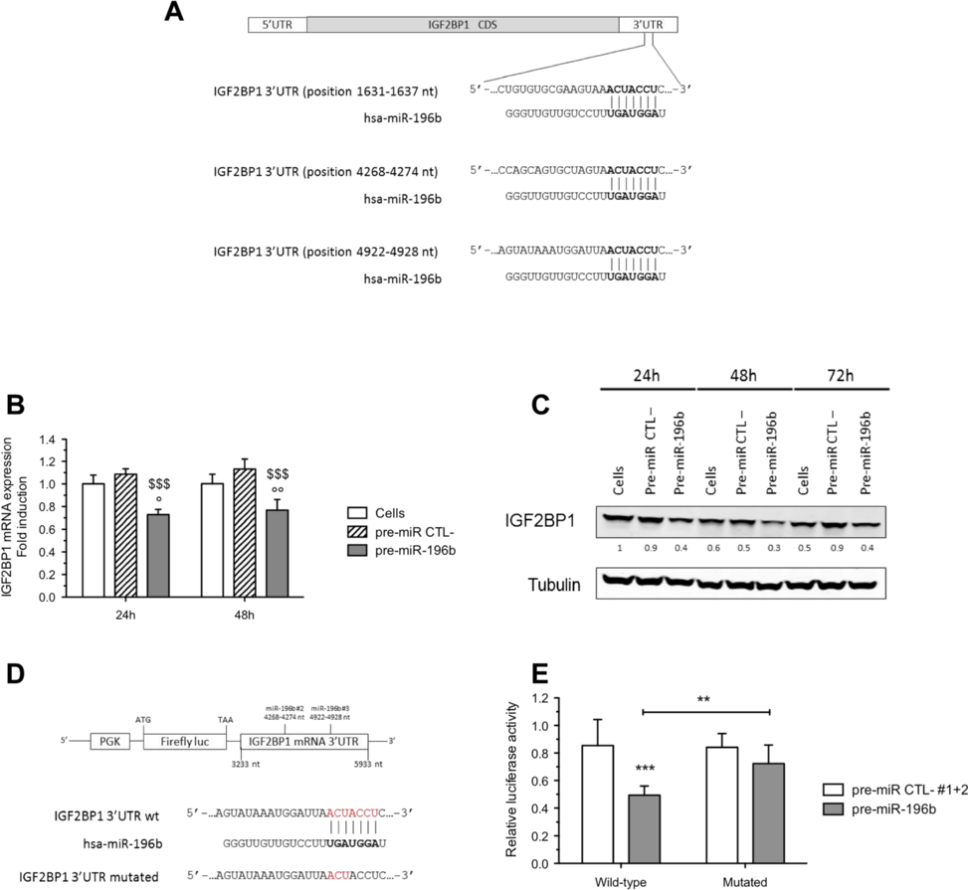
**IGF2BP1 is a direct target of miR-196b in HepG2 cells. (A)** The putative miR-196b targeted sequence in the *igf2bp1* gene. TargetScan6.2 predicts three binding sites in IGF2BP1 3′-UTR. The alignment of the seed region of miR-196b with 3′UTR is shown. **(B)** Expression level of IGF2BP1 mRNA in the pre-miR-196b transfected cells determined by RT-qPCR was down-regulated in comparison to pre-miR negative control transfected cells (pre-miR CTL-) or untransfected cells (Cells), 24 or 48 h post-transfection (means ± 1 SD, n = 3). °, °°: significantly different from untransfected cells (p < 0.05, p < 0.01), $$$: significantly different from pre-miR negative control transfected cells (p < 0.001), for each group (N, H, NE, HE) respectively. **(C)** Protein abundance of IGF2BP1 in the pre-miR-196b transfected cells determined by western blot was down-regulated in comparison to pre-miR negative control transfected cells (pre-miR CTL-) or untransfected cells (Cells), 24, 48 and 72 h after transfection. Numbers correspond to the quantification of the abundance of protein of interest normalized to the abundance of α-tubulin. **(D)** Schematic representation of the seed region match between miR-196b and the putative IGF2BP1 3′UTR. The mutation of five nucleotides in the seed region is shown. **(E)** pmiRGLO luciferase reporters containing either the wild-type or the mutant (mutated) human IGF2BP1 3′UTR were co-transfected into HepG2 cells with pre-miR negative control or pre-miR-196b (50 nM) during 72 h. 72 h post-transfection, the cells were assayed using a dual luciferase assay. Firefly luciferase values were normalized to Renilla luciferase values and plotted as relative luciferase activity (means ± 1 SD, n = 8). **: significantly different from wild-type reporter (p < 0.01), ***: significantly different from pre-miR negative control transfected cells (p < 0.001).

TargetScan 6.2 revealed 3 putative miR-196b seed match sites present in the 3′UTR of IGF2BP1 mRNA (Figure 4A). IGF2BP1 is a RNA-binding protein that controls the localization, translation and/or turnover of specific target transcripts [22]. To experimentally validate that IGF2BP1 is an endogenous target of miR-196b, HepG2 cells were transfected with pre-miR-196b or pre-miR negative control and the IGF2BP1 mRNA and protein levels were measured by RT-qPCR and western blot, respectively (Figure 4B-4C). As shown in Figure 4B, IGF2BP1 mRNA abundance was significantly reduced by miR-196b overexpression compared to cells transfected with negative control 24 and 48 h after transfection. Similarly, the abundance of IGF2BP1 protein was decreased by miR-196b overexpression when compared to pre-miR negative control 24, 48 and 72 h after the transfection (Figure 4C). Even if miRNAs can act mainly through translational repression rather than mRNA degradation, our data showed that miR-196b regulates both IGF2BP1 mRNA and protein expression levels. These results suggest that IGF2BP1 could be a direct target of miR-196b.

To demonstrate that the negative regulatory effects of miR-196b exerted on IGF2BP1 expression were mediated through the binding of miR-196b to the predicted sites in the 3′UTR of IGF2BP1 mRNA, a reporter plasmid (pmiRGLO IGF2BP1 3′UTR) containing a part of IGF2BP1 3′UTR which includes 2 predicted binding site (out of 3 sites), downstream of the firefly luciferase reporter plasmid, was used (Figure 4D). The reporter plasmid and pre-miR negative control (or pre-miR-196b) were co-transfected in HepG2 cells. As expected, miR-196b overexpression resulted in a significant decrease in the luciferase reporter activity compared to cells transfected with pre-miR negative control (Figure 4E). Furthermore, a mutated reporter plasmid containing 3 nucleotide mutations in the miR-196b seed match sites in the IGF2BP1 mRNA 3′UTR was used (Figure 4D). In contrast to the wild-type reporter plasmid, miR-196b had no significant effect on the reporter luciferase activity of the mutated plasmid, indicating that miR-196b interacts directly with 3′UTR of IGF2BP1 (Figure 4E). These results demonstrated that miR-196b directly targets the 3′UTR of IGF2BP1 mRNA leading to the down-regulation of its expression.

Taken together, proteomic analysis, western blot, RT-qPCR and luciferase activity data provide strong evidence that IGF2BP1 mRNA is a direct target of miR-196b.

### miR-196b overexpression has the same effects than the IGF2BP1 down-regulation on cell proliferation and apoptosis

To study effects of IGF2BP1 down-regulation induced by miR-196b, HepG2 cells were transfected with either pre-miR-196b or with IGF2BP1 siRNA and cell viability, proliferation and apoptotic profile were evaluated in normal culture conditions.

**Figure 5:**
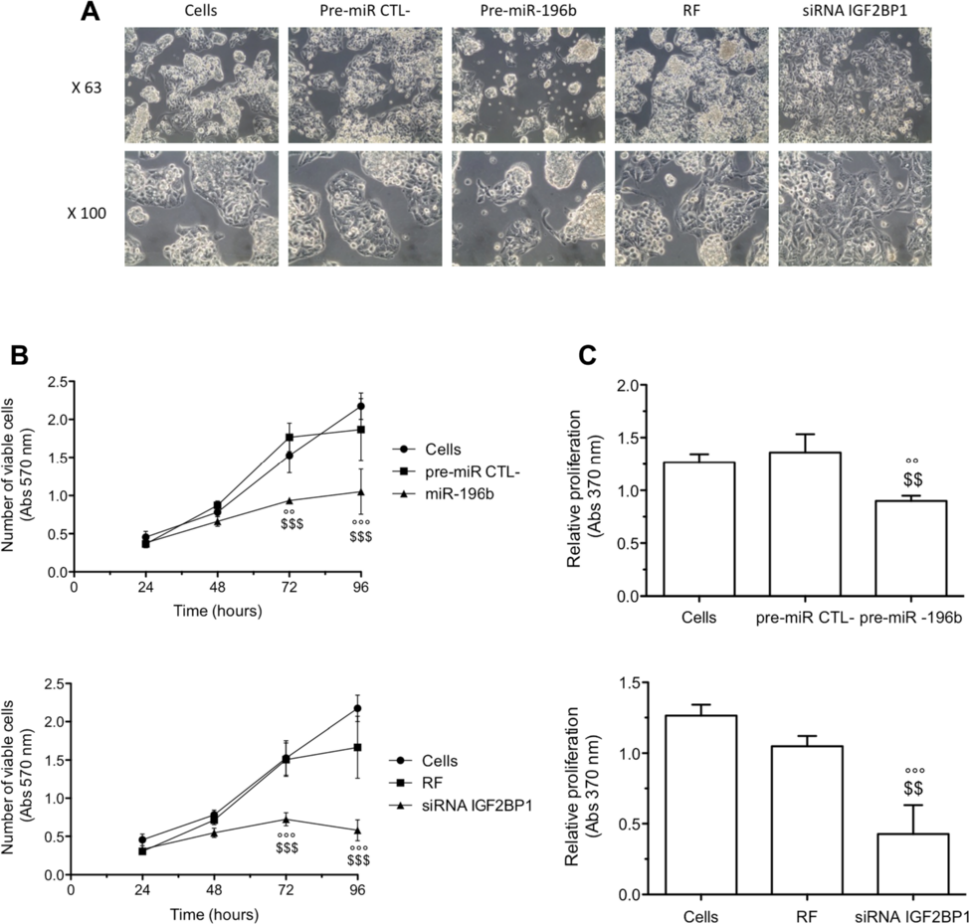
**Effects of miR-196b overexpression or IGF2BP1 silencing on cell morphology, viability and proliferation.** HepG2 cells were transfected either with pre-miR negative control (pre-miR CTL-) or pre-miR-196b (50 nM) either with RISC Free negative control (RF) or IGF2BP1 siRNA (50 nM). **(A)** 72 h after transfection, the cell morphology was observed by phase contrast microscopy. **(B)** Cell viability assay (MTT assay) was performed 24, 48, 72 and 96 h after transfection of HepG2 cells (means ± 1 SD, n = 3). **(C)** Cell proliferation assay (BrdU ELISA assay) was performed 72 h after transfection (means ± 1 SD, n = 3). °°, °°°: significantly different from untransfected cells (Cells) (p < 0.01, p < 0.001), $$, $$$: significantly different from pre-miR negative control transfected cells or RF negative control transfected cells (p < 0.01, p < 0.001).

We assessed the cell morphology by phase contrast microscopy, 72 h after pre-miR-196b or IGF2BP1 siRNA transfection. miR-196b overexpression reduced the cell number and floating cells were observed. On the other hand, IGF2BP1 silencing seems to change the cell morphology since an increase in cell size was observed (Figure 5A).

The number of viable cells was then assessed using a MTT assay. Results showed that the number of untransfected cells or cells transfected with negative controls (pre-miR negative control or RF) increased with time, suggesting cell proliferation. miR-196b overexpression decreased significantly the number of viable cells by 47 and 43.7%, 72 and 96 h post-transfection, respectively. IGF2BP1 silencing also decreased the number of viable cells by 51.9 and 65.2%, 72 and 96 h post-transfection, respectively (Figure 5B).

Proliferation was then directly measured by using BrdU incorporation 72 h after transfection. Results showed that the proliferation rate was significantly lower when cells overexpressed miR-196b compared to cells transfected with pre-miR negative control (33.8% inhibition of cell proliferation). A significant decrease was also observed when cells were transfected with IGF2BP1 siRNA compared to RISC Free 72 h post-transfection (59.2% inhibition of cell proliferation) (Figure 5C).

**Figure 6:**
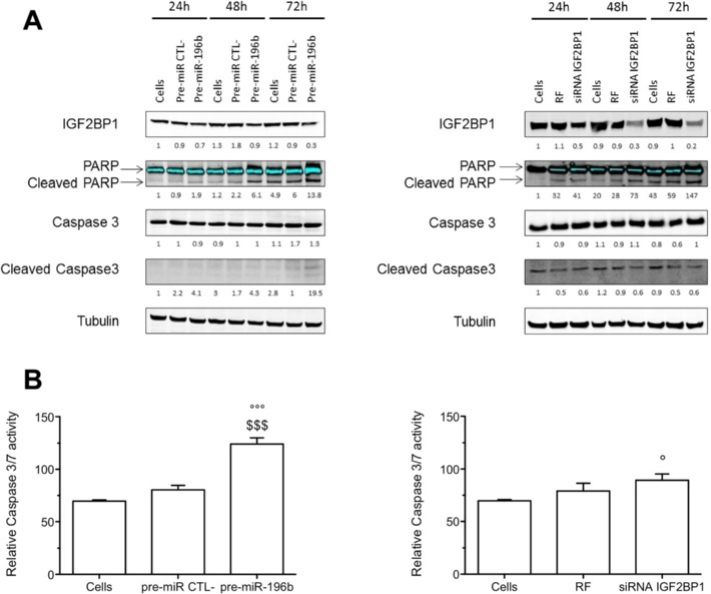
**Effects of miR-196b overexpression or IGF2BP1 silencing in regulation of apoptosis under normoxia. (A)** HepG2 cells were transfected with either pre-miR negative control (pre-miR CTL-) or pre-miR-196b (50 nM), either with RISC free negative control (RF) or siRNA IGF2BP1 for 24, 48 and 72 h. IGF2BP1, cleaved PARP and cleaved caspase 3 were detected in total cell extracts by western blotting analysis, using specific antibodies. Immunodetection of α-tubulin was used to assess the total amount of proteins loaded on the gel. Numbers correspond to the quantification of the abundance of protein of interest normalized to the abundance of α-tubulin. **(B)** 48 h after transfection, the caspase 3/7 activity was assayed by measuring free AFC released from the cleavage of the caspase 3/7 substrate Ac-DEVD-AFC. Results are expressed in fluorescence intensity, as means ± 1 SD (n = 3). °, °°°: significantly different from untransfected cells (Cells) (p < 0.05, p < 0.001), $$$: significantly different from pre-miR negative control transfected cells (p < 0.001).

We then assessed apoptosis by studying PARP and caspase 3 cleavage and measuring caspase 3/7 activity assay. As already shown in Figure 4C, miR-196b overexpression decreased IGF2BP1 protein abundance. In addition, miR-196b overexpression induced an increase in PARP cleavage 48 and 72 h post-transfection (Figure 6A, left panel). However, even if we did not detect an increase in caspase 3 cleavage by western blot, miR-196b overexpression induced a significant increase in caspase 3/7 activity by 54%, 48 h post- transfection (Figure 6B). To evaluate if IGF2BP1 is involved in the pro-apoptotic effects of miR-196b, IGF2BP1 expression was silenced using siRNA. As shown in Figure 6A (right panel), IGF2BP1 silencing strongly decreased IGF2BP1 protein abundance 24, 48 and 72 h after transfection (56, 72 and 80% of inhibition, respectively). The inhibition induced an increase in PARP cleavage but not in caspase 3 cleavage. In parallel, IGF2BP1 silencing induced a significant increase in caspase 3/7 activity by 13% but to lower extent than the one induced by miR-196b overexpression (Figure 6B, right panel). These results suggest that miR-196b promoted apoptosis and that IGF2BP1 contributes at least in part to the pro-apoptotic action of miR-196b.

Taken together, these results showed that IGF2BP1 silencing as well as miR-196b overexpression had similar effects resulting in cell proliferation inhibition and in apoptosis initiation in HepG2 cells.

### IGF2BP1 silencing but not miR-196b overexpression decreases myc mRNA expression

**Figure 7:**
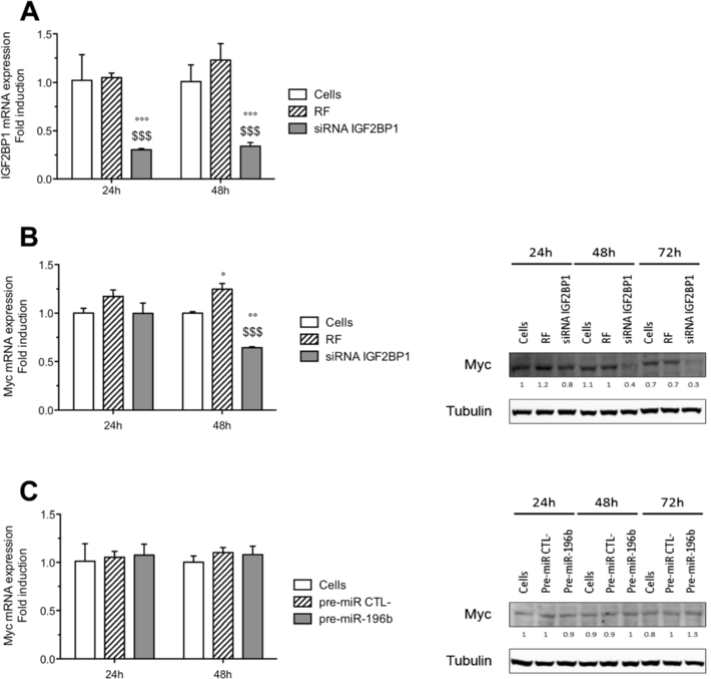
**Effects of miR-196b overexpression or IGF2BP1 silencing on c-myc mRNA expression and protein level. (A)** HepG2 cells were transfected with RISC free negative control (RF) or siRNA IGF2BP1 (50 nM) during 24 or 48 h and the mRNA expression level of IGF2BP1 was determined by RT-qPCR (means ± 1 SD, n = 3). °, °°, °°°: significantly different from untransfected cells (Cells) (p < 0.05, p < 0.01, p < 0.001), $$$: significantly different from RF negative control transfected cells (p < 0.001) for each group 24 or 48 h respectively. **(B)** HepG2 cells were transfected with RISC free negative control (RF) or siRNA IGF2BP1 (50 nM) during 24, 48 and 72 h and the mRNA expression level and the protein abundance of c-myc were determined by RT-qPCR and western blot respectively. **(C)** HepG2 cells were transfected with pre-miR negative control (pre-miR CTL-) or pre-miR-196b (50 nM), during 24, 48 and 72 h and the mRNA expression level and the protein abundance of c-myc were determined by RT-qPCR and western blot respectively. Numbers correspond to the quantification of the abundance of protein of interest normalized to the abundance of α-tubulin.

A major function of IGF2BP1 is the stabilization of numerous mRNAs. Among the known IGF2BP1 target mRNAs are actin and c-myc [23,24]. Myc represents an interesting target because of its role as a proto-oncogene. Furthermore, it was shown to be a target of miR-196b in endometriotic stromal cells [25]. Finally, several studies showed that IGF2BP1 promoted cell proliferation by enhancing c-myc expression [26]. Thus, the effects of IGF2BP1 silencing and of miR-196b overexpression on myc mRNA and protein levels were assessed in HepG2 cells. IGF2BP1 silencing decreased IGF2BP1 mRNA level by 70 and 66%, 24 and 48 h post-transfection respectively (Figure 7A). The silencing induced an inhibition by 48% of myc mRNA expression that was only detected 48 h after transfection (Figure 7B). Similarly, the abundance of myc protein was decreased by IGF2BP1 silencing compared to RF negative control 48 and 72 h after transfection (Figure 7B). However, miR-196b overexpression did not decrease the myc mRNA and protein levels (Figure 7C).

Taken together, results showed that miR-196b mediates its anti-proliferative and pro-apoptotic effects through a c-myc-independent pathway, while IGF2BP1 silencing might exert its anti-proliferative and pro-apoptotic effects at least in part through a c-myc-dependent pathway.

### IGF2BP1 silencing does not reverse the hypoxia-induced chemoresistance

**Figure 8:**
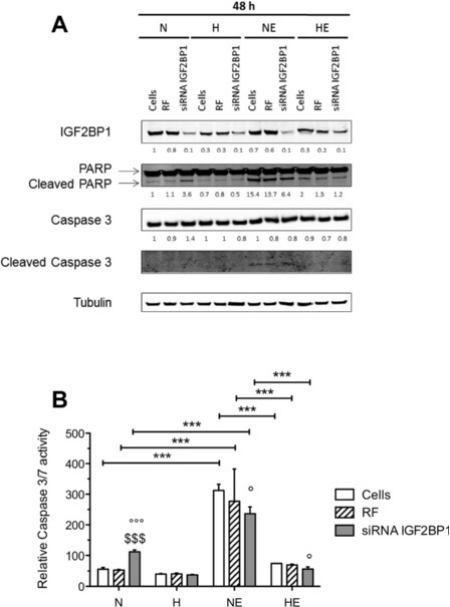
**Role of IGF2BP1 in the chemoresistance induced by hypoxia.** HepG2 cells were transfected with RISC free negative control (RF) or siRNA IGF2BP1 (50 nM) during 48 h and then cells were incubated under normoxia (N) or hypoxia (H) with or without etoposide (E) (50 μM) during 16 h. **(A)** IGF2BP1, cleaved caspase 3 and cleaved PARP were detected in total cell extracts by western blotting analysis, using specific antibodies. Immunodetection of α-tubulin was used to assess the total amount of proteins loaded on the gel. Numbers correspond to the quantification of the abundance of protein of interest normalized to the abundance of α-tubulin **(B)** 48 h after transfection, the caspase 3/7 activity was assayed by measuring free AFC released from the cleavage of the caspase 3/7 substrate Ac-DEVD-AFC. Results are expressed in fluorescence intensity, as means ± 1 SD (n = 3). ***: (p < 0.001), °, °°°: significantly different from untransfected cells (Cells) (p < 0.05, p < 0.001), $$$: significantly different from pre-miR negative control transfected cells (p < 0.001) for each group N, H, NE, HE respectively.

Since we demonstrated that IGF2BP1 is a target of miR-196b and because miR-196b was shown to modulate hypoxia-induced chemoresistance, we sought whether the chemoresistance induced by hypoxia is due to IGF2BP1 expression inhibition. For that, HepG2 cells were transfected with IGF2BP1 siRNA for 48 h and then incubated under normoxia or hypoxia with or without etoposide, and apoptotic markers were studied by western blot (Figure 8A). IGF2BP1 silencing decreased IGF2BP1 protein abundance in cells incubated under normoxia or hypoxia with or without etoposide 48 h post-transfection. However, IGF2BP1 silencing did not modify the chemoresistance induced by hypoxia. Indeed, no increase in PARP and caspase 3 cleavage was observed. These data were confirmed using a caspase 3/7 activity assay that showed no modification of caspase 3/7 activity when cells were transfected with IGF2BP1 siRNA (Figure 8B).

These results indicated that IGF2BP1 silencing alone is not sufficient to reverse the hypoxia-induced chemoresistance probably because other miR-196b targets could be involved.

## Discussion

Previously we, and others, have observed that hypoxia protects cancer cells from the death induced by chemotherapies [7-10]. Our study provided new data indicating that some of miRNAs induced/inhibited by hypoxia are involved in chemoresistance. The most well-known of miRNAs identified as a master regulator of hypoxia response is miR-210 [18,20]. In this study, we confirmed that miR-210 was up-regulated under hypoxia. Furthermore, results showed that miR-210 had an anti-apoptotic effect. Indeed, the overexpression of miR-210 in HepG2 cells under normoxia protected HepG2 cells from apoptosis induced by etoposide. These results are in agreement with other studies showing that miR-210 inhibits apoptosis and protects cancer cells from cell death [27-30].

Here, for the first time, we identified a new miRNA down-regulated by hypoxia: miR-196b. miR-196b is a member of the miR-196 sub-family, which comprises miR-196a and miR-196b. miR-196b differs from miR-196a by one nucleotide [31]. miR-196b is associated with some types of leukemia but very few data exist on the role of miR-196b in solid tumors. Our results showed that hypoxia significantly decreased miR-196b expression induced by etoposide in HepG2 cells. Several studies have demonstrated that miR-196b has a tumor suppressive function [25,32]. Here, we demonstrated the importance of miR-196b for modulating the hypoxia-induced chemoresistance.

In order to understand the contribution of miR-196b in chemoresistance induced by hypoxia, it is important to identify its targets. It is well known that miRNAs regulate biological processes by inhibiting gene expression by translational repression rather than mRNA degradation in mammalian cells [33]. It is why we used a proteomic approach to identify new direct targets. Among spots up- and down-regulated by miR-196b overexpression, 172 proteins were identified. Based on computational predictions and results obtained with a proteomic approach, 8 proteins were identified as potential targets of miR-196b. We focused on IGF2BP1, a member of the RNA-binding proteins [22]. Among several functional activities, IGF2BP1 binds mRNAs, prevents miRNA-mediated repression and regulates tumor progression [34]. IGF2BP1 was identified as a target of several miRNAs (miR-494, let-7, miR-625) [35-37]. We have demonstrated that miR-196b overexpression significantly decreased the mRNA and protein levels of IGF2BP1. In order to validate that IGF2BP1 is a direct target of miR-196b, we used luciferase activity reporters. The results validated that miR-196b binds the 3′UTR of IGF2BP1 mRNA inducing a decrease in mRNA and protein levels. To the best of our knowledge, this is the first time that IGF2BP1 is shown to be a direct target of miR-196b.

We then investigated whether IGF2BP1 is involved in chemoresistance. Indeed, some miRNAs directly influence the sensitivity of tumor cells to anticancer agents. Such an example is the let-7 family of miRNAs that inhibits Bcl-xl expression and potentiates sorafenib-induced apoptosis in human hepatocarcinoma [16]. Here, we have shown that IGF2BP1 depletion in cells incubated under hypoxia in the presence of etoposide did not induce apoptosis. These results indicate that miR-196b might regulate the hypoxia-induced chemoresistance by the regulation of other targets than IGF2BP1 or that inhibition the expression of one protein is not enough to recapitulate the whole effect of miR-196b. The expression of other anti-apoptotic proteins could be regulated by miR-196b. In addition, the overexpression of a combination of miR-196b and other miRNAs (e.g. miR-210) in order to reverse the chemoresistance to etoposide induced by hypoxia could be the object of future studies.

On the other hand, miR-196b overexpression and IGF2BP1 silencing displayed similar effects on cell proliferation and apoptosis under normal culture conditions, by significantly decreasing the proliferation and inducing the apoptosis in HepG2 cells, suggesting an anti-tumor role of the miR-196b/IGF2BP1 pathway. These results are in agreement with recent studies performed in different subtypes of cancer cells suggesting a general mechanism of pro-apoptotic and anti-proliferative effects of miR-196b. For example, Abe et al. have shown that miR-196b decreased cell proliferation and induced apoptosis in endometriotic stromal cells [25]. Yue Liu et al. evidenced that miR-196 overexpression decreased the proliferation in K562 cells [38]. Furthermore, miR-196b overexpression significantly reduced cell viability, clonogenicity, proliferation and invasion in cervical cancer [32]. In parallel to miR-196b overexpression effects, we show that the IGF2BP1 silencing also reduced cell proliferation and promoted apoptosis. These results are in agreement with two recent studies. First, Mongroo et al. have shown that the IGF2BP1 silencing plays a role in modulating cell survival by promoting PARP and caspase 3 cleavage in SW480 cells [39]. Secondly, a recent study showed that the IGF2BP1 depletion by siRNA reduces the proliferation and induces apoptosis in hepatocarcinoma cells [26]. More interestingly, several studies reported that IGF2BP1 plays a major role in liver carcinogenesis and in hepatocarcinoma progression [26,37].

IGF2BP1 silencing resulted in cell morphology changes that differ from those caused by miR-196b overexpression*.* Two other works reported changes in cell morphology upon IGF2BP1 silencing. The transient knockdown of IGF2BP1 induced an increase in size and apparent flattening of adherent HEK293 cells. Results suggested that the shift in cell size was due to altered cytoskeletal organization rather than an overall increase in cell mass. In parallel, IGF2BP1 silencing was shown to decrease mesenchymal-like cell properties by diminishing the expression of the transcriptional regulators LEF1 and SLUG (SNAI2). IGF2BP1 silencing also decreased cell migration [40]. IGF2BP1 was also shown to regulate the formation of lamellipodia and invadopodia. In osteosarcoma-derived U2OS cells, knockdown of IGF2BP1 induced a severe loss of stress fibers and an increase in the number of needle-like F-actin structures thus impairing cell migration. IGF2BP1 decreased the motility of these cells by targeting mRNAs encoding MAPK4 and PTEN [41]. IGF2BP1 also targets ß-actin encoding mRNA thus regulating cell adhesion and spreading [22]. The reason why the change in cell morphology was observed upon IGF2BP1 silencing but not caused by miR-196b overexpression is unclear. Two hypotheses can be proposed: either the decrease in IGF2BP1 protein level induced by miR-196b overexpression was not strong enough to affect IGF2BP1 targets to a level low enough to observe its consequences; or miR-196b has other mRNA targets whose inhibition counteracts the effect of the inhibition of IGF2BP1 expression.

IGF2BP1 has been shown to be involved in tumor development by targeting mRNAs such as IGF2 and c-myc [42]. In addition, several reports have described that IGF2BP1 promotes tumor cell proliferation by enhancing the expression of c-myc [26,43]. Indeed, IGF2BP1 is a mRNA-binding protein that acts in cytoplasm by controlling the localization, translation and/or turnover of specific transcripts. For example, IGF2BP1 silencing was shown to induce elevated degradation of c-myc mRNA [43]. Due to fact that c-myc is a proto-oncogene, a regulator of cell proliferation and apoptosis and that c-myc is a possible target of miR-196b based on bioinformatics algorithms [44], we decided to investigate whether c-myc is involved in anti-proliferative and pro-apoptotic effects of miR-196b and IGF2BP1 silencing or not. IGF2BP1 silencing reduced c-myc expression in HepG2 cells, but no modification has been observed in response to miR-196b overexpression. Hence, miR-196b overexpression and IGF2BP1 silencing induced anti-proliferative and pro-apoptotic effects but these effects might be myc-independent in cells that overexpress miR-196b. These results differ from results of Abe et al. and Bhatia et al. who showed that miR-196b overexpression in endometriotic cyst stromal cells [25] and in B-cell acute lymphoblastic leukemia [44] resulted in the down-regulation of c-myc. While the abundance of mRNA might be very different in the cell types used in the different studies, one could also suggest, even if all cell types are from human origin, that the region in the 3′UTR of the mRNA, recognized by the miR-196, might be differentially occupied by proteins binding mRNA in these regions. The accessibility for the miR-196 might thus be affected and thus functional impact of the miR-196 on the regulation of c-myc expression could be different in our study and results previously published.

Taken together, these results suggest that miR-196b anti-proliferative and pro-apoptotic effects type in normal condition culture can be myc-dependent or not, depending on the cell.

## Conclusions

In conclusion, these results evidence the essential role of miRNAs in chemoresistance induced by hypoxia but did not allow the identification of a unique target able to reverse this chemoresistance. Indeed, considering the fact that miRNAs can target several mRNAs simultaneously, we further established that besides the fact that IGF2BP1 is a direct target of miR-196b, the apoptosis promoting effect of miR-196b is not mainly through targeting IGF2BP1. However, IGF2BP1 is a functional target of miR-196b involved in regulating HepG2 cell viability and proliferation in normal culture conditions.

## Methods

### Cell culture and hypoxia incubation

Human hepatoma HepG2 cells were maintained in culture in 75-cm^2^ polystyrene flasks (Costar, Lowell, MA, USA) with D-MEM low glucose (Invitrogen, Carlsbad, CA, USA) containing 10% of fetal calf serum (FCS) (Invitrogen) and incubated under an atmosphere containing 5% CO_2_. For hypoxia experiments (1% O_2_), cells were incubated in serum-free CO_2_-independent medium (Invitrogen, cat n°18045) supplemented with 0.5 mM L-glutamine (Sigma, Saint Louis, USA) with or without 50 μM etoposide (Sigma). Normoxic control cells were incubated in the same conditions but kept in normal atmosphere (21% O_2_).

### TaqMan Low density array

At the end of 16 h incubation under normoxia or hypoxia with or without etoposide, total RNA was extracted from cells using the Total RNAgent extraction kit (Promega, Madison, WI, USA). mRNA contained in 2 μg total and miRNA was reverse transcribed using the “High Capacity cDNA Archive” kit (Applied Biosystems, Carlsbad, CA, USA) according to the manufacturer’s instructions. An amount corresponding to 100 ng of retrotranscribed total RNA in 50 μl was then mixed with 50 μl of the “TaqMan Universal PCR master Mix” (Applied Biosystems) and loaded into one of the 8 fill ports the microfluidic array. “TaqMan Array Human MicroRNA panel v1.0” (Applied Biosystems) is microfluidic card that contains assays for the detection of 365 miRNAs in addition to 2 endogenous controls (RNU44 and RNU48). miRNA expression level was quantified using the threshold cycle method [45].

### Total and/or miRNA extraction

miRNA were extracted from samples containing total RNA with TRI Reagent Solution (Invitrogen) and the miRNeasy mini kit (Qiagen, Hilden, Germany) following manufacturer’s instructions. Total RNA without miRNA were extracted with RNeasy mini kit (Qiagen) following manufacturer’s instructions. RNA concentration was determined by using Nanodrop.

### Reverse transcription and quantitative real-time PCR for miRNA expression

miRNA expression was evaluated using TaqMan MicroRNA Assay (Applied Biosystems) as specified in manufacturer’s instructions. Briefly, single‑stranded cDNA was synthesized by the TaqMan MicroRNA Reverse Transcription Kit from 200 ng of total RNA in a 15 μl reaction. The reactions were incubated sequentially at 16°C for 30 min, at 42°C for 30 min and then at 85°C for 5 min. cDNAs were then amplified by quantitative PCR in a 7900HT Sequence Detection System using the TaqMan microRNA Assays Human Panel sequence‑specific primers. The 20 μl PCR included 10 μl of 2x Universal PCR Master Mix (No AmpErase, UNG), 1 μl of each 20x TaqMan MicroRNA Assay Mix and 1.5 μl of reverse transcription product. The reaction mixtures were incubated in a 96‑well plates at 95°C for 10 min, followed by 40 cycles of 15 sec at 95°C, and 1 min at 60°C. The expression level of each miRNA was measured using the threshold cycle method using the expression of RNU44 (endogenous control) as reference and the expression of miRNAs under normoxia as calibrator. All reagents were supplied by Applied Biosystems.

### Reverse transcription and quantitative real-time PCR for total RNA

cDNA was synthesized with 2 μg of total RNA using SuperScript II Reverse Transcriptase (Roche, Basel, Switzerland) and random hexamers primers for IGF2BP1 gene or oligodT for Myc gene according to the manufacturer’s instructions. Amplification reaction assays contained 1x SYBR Green PCR Mastermix (Applied Biosystem) and primers (IDT, Coralville, IA, USA) at the optimal concentrations. Primers for detection of mRNA were as follows IGF2BP1: 5′-CAGGAGATGGTGCAGGTGTTTATCC-3′ and 5′-GTTTGCCATAGATTCTTCCCTGAGC-3′, c-myc: 5′-CAGCTGCTTAGACGCTGGATT-3′ and 5′-GTAGAAATACGGCTGCACCGA-3′ [26]. The RNA expression level was measured using the threshold cycle method [45] using 23 kDa as the normalization reference.

### miRNA transfection

Chemically synthesized miRNA duplexes, called pre-miR-210 and pre-miR-196b and negative control miRNA (pre-miR CTL-), were purchased from Ambion (Life Technologies, Carlsbad, CA, USA). HepG2 transfection was performed in 6-well plates for caspase 3/7 activity assay (50 000 cells per well) or in 25-cm^2^ polystyrene flasks for protein extraction (750 000 cells per flask) with Dharmafect 1 transfection reagent used at a 1:500 dilution (Dharmacon, Lafayette, CO, USA) and with pre-miR at a final concentration of 50 nM. Cells were incubated for 24 h with transfection medium. The medium was then replaced by fresh medium with 10% of FCS at least 6 h before being incubated under hypoxia. Hypoxia and/or etoposide exposure during 16 h was performed 24 or 48 h after transfection.

### siRNA transfection

IGF2BP1 silencing was achieved using the following siRNA sequence: 5′-CCGGGAGCAGACCAGGCAA(dTdT)-3′. RISC-free (RF) siRNA purchased from Dharmacon was used as negative control. For siRNA experiments, 750 000 cells were seeded in 25 cm^2^ polystyrene flasks (Costar). The next day, cells were transfected with 50 nM siRNA using Dharmafect 1 (Dharmacon) transfection reagent according to manufacturer’s instructions. The transfection medium was removed 24 h after transfection and replaced by fresh culture medium. Cells were then incubated for 24, 48, 72 and 96 h and then incubated under normoxia or hypoxia with or without etoposide for 16 h.

### Western blot analysis

HepG2 cells, seeded in 25 cm^2^ flasks, were scrapped in 200 μl of lysis buffer (40 mM Tris pH 7.5, 150 mM KCl, 1 mM EDTA, 1% triton X-100) containing a protease inhibitor mixture (« Complete » from Roche Molecular Biochemicals, 1 tablet in 2 ml H_2_O, added at a 1: 10 dilution) and phosphatase inhibitors (25 mM NaVO_3_, 250 mM PNPP, 250 mM α-glycerophosphate and 125 mM NaF, at a 1: 10 dilution). Proteins were separated by SDS-PAGE on 4–12% NuPAGE Bis-Tris gels with MES or MOPS buffer (Invitrogen) and then transferred onto a low fluorescence background polyvinylidene fluoride (PVDF) membrane (Immobilon-FL, Millipore, Billerica, MA, USA). For fluorescence detection, membranes were blocked 1 h at room temperature in Odyssey blocking buffer (BD Biosciences, Franklin Lakes, MJ, USA) diluted 2X in PBS and then overnight at 4°C in Odyssey blocking buffer with 0.1% Tween containing a specific antibody: mouse anti-PARP-1 monoclonal antibody (#556493 from BD Pharmingen, Franklin Lakes, MJ, USA), rabbit anti-caspase3 antibody (#9662 from Cell Signaling, Dancer, MA, USA), rabbit anti-IGF2BP1 antibody (#2852 from Cell Signaling), mouse anti-Myc (#sc-42 from SantaCruz Technologies, Dallas, TX, USA) and mouse anti-α-tubulin monoclonal antibody (#T5168 from Sigma-Aldrich). After 3 washes of 10 min in PBS-0.1% Tween, the incubation with the secondary antibody (goat anti-rabbit or anti-mouse IR Dye labelled antibody 1/7 500 dilution) (Licor, Lincoln, NE, USA,) was performed for 1 h in Odyssey blocking buffer with 0.1% Tween followed by 3 washes of 10 min in PBS-Tween and 2 washes in PBS. The membrane was then dried 30 min at 37°C and scanned using Odyssey infrared imaging system (Licor). Immunodetection of α-tubulin was used as the loading control.

### Caspase-3/7 activity assay

The fluorogenic substrate Ac-DEVD-AFC (BD Pharmingen) was used to measure caspase-3/7 activity according to Lozano et al. [46]. Protein extraction and the assay for caspase-3/7 activity were performed according to Notte et al. [47] using 20 μg of protein extracts.

### Subcellular fractionation

HepG2 cells were seeded at a density of 5 500 000 cells in 75-cm^2^ flasks. The next day, cells were then transfected with pre-miR negative control or pre-miR-196b (50 nM) for 24 h. Cells were harvested in 0.25 M saccharose containing a protease inhibitor mixture (Roche Molecular Biochemicals) and phosphatase inhibitors (25 mM NaVO_3_, 250 mM PNPP, 250 mM α-glycerophosphate and 125 mM NaF, at a 1: 10 dilution) and homogenized by 5 strokes of a B plunger in a Dounce. The homogenate was centrifuged for 10 min at 1 000 rpm (Labofuge 400R Heraeus Instruments). The pellet was resuspended in 0.25 M saccharose and named the N (Nuclear) fraction. The supernatant was recovered and centrifuged at 8 000 rpm (Beckman L7-35, rotor 50 Ti) for a duration dependent on the volume. The pellet was resuspended in 0.25 M saccharose and named the MLP fraction. The supernatant was centrifuged at 35 000 rpm (Beckman L7-35, rotor 50 Ti) for a duration dependent on the volume. The supernatant was recovered and named the S fraction, which contains the cytosolic proteins.

### Two-dimensional electrophoresis (2-DE) analysis and protein identification

Labeling with the cyanine dyes (GE Healthcare, Uppsala, Sweden) was performed as described in details in the supplementary methods (see Additional file 2). An amount corresponding of 25 μg of each individual labeled samples (pre-miR CTL-, pre-miR-196b and pooled standard samples from four replicate experiments) were mixed and subjected to isoelectric focusing on a IPGphor isoelectric focusing unit (GE Healthcare) along a continuous 3–11 pH gradient using IPG strips (18 cm, nonlinear 3 – 11 pH, GE Healthcare) (details in supplementary methods, see Additional file 2). Gels were scanned with a Typhoon 9400 imager (GE Healthcare) to detect cyanin labeled proteins. Determination of protein spot abundance and statistical analyses were carried out automatically using the DeCyder 2D Differential Analysis software 6.0 (GE Healthcare). Protein spots with more than 1.2-fold change in volume after normalization (between pre-miR CTL- and pre-miR-196b in the 4 replicates) and statistically significant Student’s *t*-test (p < 0.05) were considered as differentially abundant.

Each spot of interest was excised automatically using the Ettan spot picking system (GE Healthcare). Detailed trypsin digestion and mass spectrometry procedures by using nanoLC-MS-MS maXis UHR-TOF (ultra-high resolution time-of-flight) coupled with a 2D-LC Dionex UltiMate 3 000 (Bruker, Bremen, Germany) are given in the section of the supplementary methods (see Additional file 2).

### miRNA target prediction

A biocomputational approach was used based on a stepwise guide to identify miR‑196b targets. We focused on mRNA predicted by either TargetScan 6.2 [48], Microcosm [49] or miRDB [50] softwares.

### Reporter constructs and dual luciferase assay

The pmiRGLO plasmid vector contains cDNA sequences encoding the *Firefly* luciferase (F-luc) reporter gene and the *Renilla* luciferase (R-luc) gene, which acts as an internal control reporter. The pmiRGLO IGF2BP1 3′UTR wild-type and mutated were kindly obtained from Yang lab [51]. The wild-type 2 701-bp fragment contains two of three putative miR-196b seed match sites (4 268 – 4 274 nt and 4 922–4 928 nt). The pmiRGLO IGF2BP1 3′UTR mutated contains mutations at 2 sites cloned into the pmiRGLO vector. One day before the transfection, HepG2 cells were seeded at 30 000 cells per well into 24-well plates. The next day, HepG2 cells were co-transfected with 0.1 μg of pmiRGLO IGF2BP1 3′UTR wild-type or mutated and either pre-miR negative control or pre-miR-196b (50 nM final concentration). Transfection was performed with Lipofectamine 2000 reagent (Invitrogen) according to the co-transfection manufacturer’s protocol. At 72 h post-transfection, firefly and renilla luciferase activities were measured using Dual-luciferase Reporter Assay System (Promega, Madison, WI, USA) according the manufacturer’s protocol. The F-luc activity was normalized to the R-luc activity.

### MTT assay

The methylthiazol tetrazolium (MTT, Sigma-Aldrich) spectrophotometric dye assay was used to detect the number of viable cells. HepG2 cells were seeded at 20 000 cells per well into 24-well plates 24 h before transfection. Cells were transfected for 24 h with either pre-miR CTL- or pre-miR-196b (50 nM) either RISC Free negative control (RF) or IGF2BP1 siRNA (50 nM) with Dharmafect. 24, 48, 72 and 96 h post-transfection, cells were incubated at 37°C for 2 h with 500 μL MTT solution (2.5 mg/mL; Sigma-Aldrich). Culture medium was then removed and formazan crystals in the cells were solubilized using lysis buffer (30% SDS, N,N-dimethyl-formamide; pH 4.7) with plate shaking for 2 h. Absorbance was measured at 570 nm.

### BrdU ELISA kit

The BrdU Cell Proliferation ELISA kit (Roche) has been used to measure cell proliferation according to manufacturer’s protocol. HepG2 cells were seeded at 2 500 cells per well into 96-well plates. 48 h after transfection, the BrdU solution (10 μM) was added during 24 h at 37°C. Absorbance was measured at 370 nm (reference wavelength 492 nm).

### Statistical analysis

SigmaStat software (Jandle Scientific, Germany) was used for the statistical analysis. Data are presented as means ± standard deviation (SD) and were evaluated by two-way ANOVA, using the Holm-Sidak method. For some analyses values did not follow a Gaussian distribution. In order to deal with this absence of normality, statistical analyses were performed on log-transformed data. In order to facilitate interpretation, untransformed data are shown.

## Additional files

Additional file 1:
**List of proteins in the cytosolic fraction identified to be differentially expressed in HepG2 cells transfected with pre-miR-196b in comparison to cells transfected with the pre-miR negative control.**
Additional file 2:
**Supplementary methods.**

## Abbreviations

IGF2BP1: Insulin-like growth factor 2 mRNA binding protein 1
miR: microRNA
PARP: Poly-(ADP-ribose) polymerase
RF: RISC Free
TLDA: TaqMan low density array
UTR: Untranslated region
