# Supplementary material for: miRNA-196b inhibits cell proliferation and induces apoptosis in HepG2 cells by targeting IGF2BP1
Source: Mol Cancer. 2015 Apr 8;14:79. doi: 10.1186/s12943-015-0349-6 (PMC4403945; doi:10.1186/s12943-015-0349-6)
Supplement: Additional file 1: — List of proteins in the cytosolic fraction identified to be differentially expressed in HepG2 cells transfected with pre-miR-196b in comparison to cells transfected with the pre-miR negative control. [file 12943_2015_349_MOESM1_ESM.docx]

**Additional file 1: List of proteins in the cytosolic fraction identified to be differentially expressed in HepG2 cells transfected with pre-miR-196b in comparison to cells transfected with the pre-miR negative control.**

For each spot one or several proteins were identified. (a) Accession number supplied by NCBI. (b) Number of unique peptides identified by Mascot and X!Tandem. (c) Ratio (%) of amino acids identified by MS analysis which corresponds to the total number of amino acids of the reference protein.

| **Fold change gel 2D** | **N° spot** | **Protein identification** | **Accession Number (a)** | **Molecular Weight** | **Number of unique peptide (b)** | **% coverage protein (c )** |
| --- | --- | --- | --- | --- | --- | --- |
| **-1,22** | **942** | IGF-II mRNA-binding protein 1 [Homo sapiens] | [Q9NZI8](http://www.uniprot.org/uniprot/Q9NZI8) | 63 kDa | 24 | 38% |
|  |  | EF1a [Homo sapiens] | [Q6IPS9](http://www.uniprot.org/uniprot/Q6IPS9) | 50 kDa | 4 | 7% |
|  |  | DEAD (Asp-Glu-Ala-Asp) box polypeptide 5 [Homo sapiens] | [P17844](http://www.uniprot.org/uniprot/P17844) | 69 kDa | 4 | 6% |
| **-1,89** | **1049** | Chaperonin containing TCP1, subunit 8 (theta) [Homo sapiens] | [P50990](http://www.uniprot.org/uniprot/P50990) | 60 kDa | 11 | 25% |
| **-1,28** | **1289** | BRISC complex subunit Abro1 [Homo sapiens] | [Q15018](http://www.uniprot.org/uniprot/Q15018) | 47 kDa | 3 | 9% |
|  |  | PRKAR1B protein [Homo sapiens] | [P31321](http://www.uniprot.org/uniprot/P31321) | 43 kDa | 3 | 13% |
|  |  | selenophosphate synthetase 2 [Homo sapiens] | [B4E093](http://www.uniprot.org/uniprot/B4E093) | 36 kDa | 2 | 8% |
|  |  | Solute carrier family 9 (sodium/hydrogen exchanger), member 3 regulator 1 [Homo sapiens] | [O14745](http://www.uniprot.org/uniprot/O14745) | 39 kDa | 2 | 8% |
| **1,18** | **2156** | Calcyclin-binding protein [Cricetulus griseus] | [G3GZL7](http://www.uniprot.org/uniprot/G3GZL7) | 21 kDa | 6 | 29% |
|  |  | RSU1 protein [Homo sapiens] | [Q15404](http://www.uniprot.org/uniprot/Q15404) | 32 kDa | 2 | 6% |
| **1,2** | **426** | methylenetetrahydrofolate dehydrogenase (NADP+ dependent) 1 | gi\|332842092 | 111 kDa | 28 | 27% |
|  |  | Staphylococcal nuclease and tudor domain containing 1 [Homo sapiens] | [Q7KZF4](http://www.uniprot.org/uniprot/Q7KZF4) | 102 kDa | 6 | 10% |
|  |  | EEF2 protein [Homo sapiens] | [P13639](http://www.uniprot.org/uniprot/P13639) | 95 kDa | 4 | 6% |
| **-1,27** | **952** | IGF-II mRNA-binding protein 1 [Homo sapiens] | [Q9NZI8](http://www.uniprot.org/uniprot/Q9NZI8) | 63 kDa | 12 | 26% |
| **1,41** | **1090** | Catalase [Homo sapiens] | [P04040](http://www.uniprot.org/uniprot/P04040) | 60 kDa | 21 | 39% |
|  |  | Phosphoribosyl pyrophosphate amidotransferase [Homo sapiens] | [A8K4H7](http://www.uniprot.org/uniprot/A8K4H7) | 57 kDa | 8 | 17% |
|  |  | UDP-glucose dehydrogenase [Homo sapiens] | [O60701](http://www.uniprot.org/uniprot/O60701) | 55 kDa | 6 | 16% |
|  |  | Pyruvate kinase, muscle [Homo sapiens] | [P14618](http://www.uniprot.org/uniprot/P14618) | 58 kDa | 5 | 13% |
|  |  | pyrroline-5-carboxylate dehydrogenase [Homo sapiens] | [P30038](http://www.uniprot.org/uniprot/P30038) | 62 kDa | 5 | 11% |
|  |  | coronin-1C [Bos taurus] | [A2VDN8](http://www.uniprot.org/uniprot/A2VDN8) | 53 kDa | 4 | 9% |
|  |  | JUB protein [Homo sapiens] | [Q96IF1](http://www.uniprot.org/uniprot/Q96IF1) | 57 kDa | 4 | 11% |
| **-1,1** | **1302** | N.D |  |  |  |  |
| **-1,45** | **2245** | N.D |  |  |  |  |
| **1,28** | **486** | Minichromosome maintenance complex component 4 [Homo sapiens] | [P33991](http://www.uniprot.org/uniprot/P33991) | 97 kDa | 29 | 44% |
|  |  | programmed cell death 6-interacting protein isoform 1 [Homo sapiens] | [Q8WUM4](http://www.uniprot.org/uniprot/Q8WUM4) | 96 kDa | 13 | 19% |
|  |  | Chain A, High Resolution Crystal Structure Of The Bovine Beta- Lactoglobulin (Isoforms A And B) In Orthorombic Space Group | [P02754](http://www.uniprot.org/uniprot/P02754) | 18 kDa | 12 | 66% |
|  |  | putative 5-methycytoisine methyltransferase [Homo sapiens] | [Q08J23](http://www.uniprot.org/uniprot/Q08J23) | 87 kDa | 12 | 22% |
|  |  | alpha S1 casein [Bos taurus] | [P02662](http://www.uniprot.org/uniprot/P02662) | 25 kDa | 11 | 47% |
|  |  | alpha-s2-like casein precursor [Bos taurus] | [P02663](http://www.uniprot.org/uniprot/P02663) | 26 kDa | 11 | 38% |
|  |  | molybdenum cofactor sulfurase [Homo sapiens] | [Q96EN8](http://www.uniprot.org/uniprot/Q96EN8) | 98 kDa | 11 | 15% |
|  |  | CSN2 protein [Bos taurus] | [P02666](http://www.uniprot.org/uniprot/P02666) | 25 kDa | 10 | 44% |
|  |  | ALB protein [Bos taurus] | [P02769](http://www.uniprot.org/uniprot/P02769) | 69 kDa | 7 | 14% |
|  |  | Aconitase 1, soluble [Homo sapiens] | [P21399](http://www.uniprot.org/uniprot/P21399) | 98 kDa | 7 | 8% |
|  |  | butyrophilin precursor [Bos taurus] | [P18892](http://www.uniprot.org/uniprot/P18892) | 59 kDa | 7 | 20% |
|  |  | lactoferrin [Bos taurus] | [B9VPZ5](http://www.uniprot.org/uniprot/B9VPZ5) | 78 kDa | 6 | 10% |
|  |  | PAS-6 and PAS-7 proteins [Bos taurus] | [Q95114](http://www.uniprot.org/uniprot/Q95114) | 48 kDa | 6 | 18% |
|  |  | Ig heavy chain precursor (B/MT.4A.17.H5.A5) - bovine | gi\|108750 (+1) | 51 kDa | 5 | 17% |
|  |  | kappa-casein [Bos taurus] | [P02668](http://www.uniprot.org/uniprot/P02668) | 21 kDa | 4 | 26% |
|  |  | polymeric immunoglobulin receptor [Bos taurus] | [P81265](http://www.uniprot.org/uniprot/P81265) | 82 kDa | 3 | 5% |
|  |  | component PP3 | gi\|741536 | 15 kDa | 2 | 22% |
|  |  | retrotransposon-derived protein PEG10 isoform 5 [Homo sapiens] | [Q86TG7](http://www.uniprot.org/uniprot/Q86TG7) | 84 kDa | 2 | 2% |
|  |  | xanthine dehydrogenase [Bos taurus] | [P80457](http://www.uniprot.org/uniprot/P80457) | 147 kDa | 2 | 3% |
|  |  | Lipoprotein lipase [Bos taurus] | [P11151](http://www.uniprot.org/uniprot/P11151) | 53 kDa | 2 | 8% |
|  |  | IGLL1 protein [Bos taurus] | [A4IFI0](http://www.uniprot.org/uniprot/A4IFI0) | 25 kDa | 2 | 14% |
|  |  | prepro complement component C3 [Bos taurus] | [Q2UVX4](http://www.uniprot.org/uniprot/Q2UVX4) | 187 kDa | 2 | 2% |
| **-1,36** | **968** | Archain [Homo sapiens] | [B0YIW5](http://www.uniprot.org/uniprot/B0YIW5) | 57 kDa | 24 | 39% |
|  |  | chaperonin containing TCP1, subunit 3 (gamma) [Homo sapiens] | [P49368](http://www.uniprot.org/uniprot/P49368) | 61 kDa | 16 | 27% |
|  |  | hCRMP-2 [Homo sapiens] | [Q16555](http://www.uniprot.org/uniprot/Q16555) | 62 kDa | 8 | 18% |
|  |  | AICAR formyltransferase/IMP cyclohydrolase bifunctional enzyme [Homo sapiens] | [P31939](http://www.uniprot.org/uniprot/P31939) | 64 kDa | 3 | 8% |
|  |  | Stress-induced-phosphoprotein 1 [Homo sapiens] | [P31948](http://www.uniprot.org/uniprot/P31948) | 63 kDa | 2 | 12% |
|  |  | Coiled-coil domain containing 6 [Homo sapiens] | [Q16204](http://www.uniprot.org/uniprot/Q16204) | 53 kDa | 2 | 6% |
|  |  | asparagine--tRNA ligase, cytoplasmic [Bos taurus] | [Q2KJG3](http://www.uniprot.org/uniprot/Q2KJG3) | 64 kDa | 2 | 5% |
| **-1,46** | **1563** | isocitrate dehydrogenase & (NADP+), soluble, isoform CRA_a (Homo spaiens) | gi 119590845 (+20) | 47 kDa | 22 |  |
|  |  | PREDICTED: 4 -hydroxyphenylpyruvate dioxygenase [ Saimiri boliviensis boliviensis) | gi 403281469 | 45 kDa | 2 |  |
| **-1,2** | **1132** | chaperonin containing t-complex polypeptide 1, delta subunit [Homo sapiens] | [P50991](http://www.uniprot.org/uniprot/P50991) | 58 kDa | 5 | 14% |
|  |  | Pyruvate kinase, muscle [Homo sapiens] | [P14618](http://www.uniprot.org/uniprot/P14618) | 58 kDa | 3 | 10% |
|  |  | UDP-glucose dehydrogenase [Homo sapiens] | [O60701](http://www.uniprot.org/uniprot/O60701) | 55 kDa | 2 | 4% |
| **-1,51** | **1007** | hCRMP-2 [Homo sapiens] | [Q16555](http://www.uniprot.org/uniprot/Q16555) | 62 kDa | 19 | 47% |
|  |  | T-complex 1 [Homo sapiens] | [P17987](http://www.uniprot.org/uniprot/P17987) | 60 kDa | 14 | 27% |
|  |  | fatty acid synthase [Homo sapiens] | [P49327](http://www.uniprot.org/uniprot/P49327) | 273 kDa | 10 | 5% |
|  |  | zinc finger protein 622 [Homo sapiens] | [Q969S3](http://www.uniprot.org/uniprot/Q969S3) | 54 kDa | 8 | 18% |
|  |  | Leukotriene A4 hydrolase [Homo sapiens] | [P09960](http://www.uniprot.org/uniprot/P09960) | 69 kDa | 5 | 11% |
|  |  | Coiled-coil domain containing 6 [Homo sapiens] | [Q16204](http://www.uniprot.org/uniprot/Q16204) | 53 kDa | 4 | 10% |
|  |  | ALB protein [Bos taurus] | [P02769](http://www.uniprot.org/uniprot/P02769) | 69 kDa | 4 | 7% |
|  |  | Archain [Homo sapiens] | [B0YIW5](http://www.uniprot.org/uniprot/B0YIW5) | 57 kDa | 3 | 7% |
|  |  | unnamed protein product [Homo sapiens] | [B2R5M8](http://www.uniprot.org/uniprot/B2R5M8) | 47 kDa | 2 | 4% |
|  |  | AICAR formyltransferase/IMP cyclohydrolase bifunctional enzyme [Homo sapiens] | [P31939](http://www.uniprot.org/uniprot/P31939) | 64 kDa | 2 | 4% |
| **1,32** | **491** | Minichromosome maintenance complex component 4 [Homo sapiens] | [P33991](http://www.uniprot.org/uniprot/P33991) | 97 kDa | 10 | 18% |
|  |  | molybdenum cofactor sulfurase [Homo sapiens] | [Q96EN8](http://www.uniprot.org/uniprot/Q96EN8) | 98 kDa | 6 | 9% |
|  |  | EEF2 protein [Homo sapiens] | [P13639](http://www.uniprot.org/uniprot/P13639) | 95 kDa | 6 | 7% |
|  |  | putative 5-methycytoisine methyltransferase [Homo sapiens] | [Q08J23](http://www.uniprot.org/uniprot/Q08J23) | 87 kDa | 4 | 5% |
|  |  | Aconitase 1, soluble [Homo sapiens] | [P21399](http://www.uniprot.org/uniprot/P21399) | 98 kDa | 3 | 3% |
|  |  | programmed cell death 6-interacting protein isoform 1 [Homo sapiens] | [Q8WUM4](http://www.uniprot.org/uniprot/Q8WUM4) | 96 kDa | 2 | 3% |
|  |  | oxysterol-binding protein 1 [Homo sapiens] | [P22059](http://www.uniprot.org/uniprot/P22059) | 89 kDa | 2 | 3% |
| **-1,53** | **1684** | Glutaredoxin 3 [Homo sapiens] | [O76003](http://www.uniprot.org/uniprot/O76003) | 37 kDa | 3 | 10% |
|  |  | Cytokine induced apoptosis inhibitor 1 [Homo sapiens] | [Q6FI81](http://www.uniprot.org/uniprot/Q6FI81) | 34 kDa | 2 | 8% |
| **-1,63** | **1137** | N.D |  |  |  |  |
| **-1,34** | **1016** | chaperonin-like protein [Homo sapiens] | [P40227](http://www.uniprot.org/uniprot/P40227) | 58 kDa | 10 | 28% |
|  |  | Phosphoglucomutase 1 [Homo sapiens] | [P36871](http://www.uniprot.org/uniprot/P36871) | 61 kDa | 9 | 19% |
|  |  | AICAR formyltransferase/IMP cyclohydrolase bifunctional enzyme [Homo sapiens] | [P31939](http://www.uniprot.org/uniprot/P31939) | 64 kDa | 5 | 14% |
|  |  | Stress-induced-phosphoprotein 1 [Homo sapiens] | [P31948](http://www.uniprot.org/uniprot/P31948) | 63 kDa | 3 | 8% |
| **1,44** | **492** | Minichromosome maintenance complex component 4 [Homo sapiens] | [P33991](http://www.uniprot.org/uniprot/P33991) | 97 kDa | 10 | 16% |
|  |  | Aconitase 1, soluble [Homo sapiens] | [P21399](http://www.uniprot.org/uniprot/P21399) | 98 kDa | 8 | 8% |
|  |  | EEF2 protein [Homo sapiens] | [P13639](http://www.uniprot.org/uniprot/P13639) | 95 kDa | 6 | 8% |
|  |  | molybdenum cofactor sulfurase [Homo sapiens] | [Q96EN8](http://www.uniprot.org/uniprot/Q96EN8) | 98 kDa | 5 | 3% |
|  |  | fatty acid synthase [Homo sapiens] | [P49327](http://www.uniprot.org/uniprot/P49327) | 273 kDa | 5 | 7% |
|  |  | putative 5-methycytoisine methyltransferase [Homo sapiens] | [Q08J23](http://www.uniprot.org/uniprot/Q08J23) | 87 kDa | 4 | 8% |
| **-1,23** | **1691** | Aldolase A, fructose-bisphosphate [Homo sapiens] | [P04075](http://www.uniprot.org/uniprot/P04075) | 39 kDa | 5 | 16% |
|  |  | Glutamic-oxaloacetic transaminase 2, mitochondrial (aspartate aminotransferase 2) [Homo sapiens] | [P00505](http://www.uniprot.org/uniprot/P00505) | 47 kDa | 2 | 6% |
| **-1,87** | **1143** | chaperonin containing t-complex polypeptide 1, delta subunit [Homo sapiens] | [P50991](http://www.uniprot.org/uniprot/P50991) | 58 kDa | 23 | 47% |
|  |  | Pyruvate kinase, muscle [Homo sapiens] | [P14618](http://www.uniprot.org/uniprot/P14618) | 58 kDa | 10 | 20% |
|  |  | Chaperonin containing TCP1, subunit 7 (eta) [Homo sapiens] | [Q99832](http://www.uniprot.org/uniprot/Q99832) | 59 kDa | 3 | 7% |
|  |  | RCC2 protein [Homo sapiens] | [A5PLK7](http://www.uniprot.org/uniprot/A5PLK7) | 50 kDa | 3 | 5% |
|  |  | CAP protein [Homo sapiens] | [D3DPU2](http://www.uniprot.org/uniprot/D3DPU2) | 52 kDa | 2 | 5% |
| **1,12** | **1840** | PDZ and LIM domain 1 [Homo sapiens] | [O00151](http://www.uniprot.org/uniprot/O00151) | 36 kDa | 17 | 58% |
|  |  | LIM and SH3 domain protein [Homo sapiens] | [Q14847](http://www.uniprot.org/uniprot/Q14847) | 30 kDa | 13 | 44% |
|  |  | aldo-keto reductase family 7, member A2 (aflatoxin aldehyde reductase) [Homo sapiens] | [O43488](http://www.uniprot.org/uniprot/O43488) | 40 kDa | 3 | 9% |
|  |  | glyceraldehyde-3-phosphate dehydrogenase [Homo sapiens] | [P04406](http://www.uniprot.org/uniprot/P04406) | 36 kDa | 2 | 6% |
|  |  | Transaldolase 1 [Homo sapiens] | [P37837](http://www.uniprot.org/uniprot/P37837) | 38 kDa | 2 | 7% |
| **1,39** | **1026** | Phosphoglucomutase 1 [Homo sapiens] | [P36871](http://www.uniprot.org/uniprot/P36871) | 61 kDa | 14 | 27% |
|  |  | arginine-tRNA-protein transferase 1-2p [Homo sapiens] | [O95260](http://www.uniprot.org/uniprot/O95260) | 58 kDa | 9 | 21% |
|  |  | Stress-induced-phosphoprotein 1 [Homo sapiens] | [P31948](http://www.uniprot.org/uniprot/P31948) | 63 kDa | 3 | 8% |
|  |  | heterogeneous nuclear ribonucleoprotein L-like [Bos taurus] | [Q08E28](http://www.uniprot.org/uniprot/Q08E28) | 60 kDa | 2 | 5% |
|  |  | Asparagine synthetase [Mus musculus] | [Q61024](http://www.uniprot.org/uniprot/Q61024) | 64 kDa | 2 | 4% |
| **-1,35** | **530** | Valosin-containing protein [Homo sapiens] | [P55072](http://www.uniprot.org/uniprot/P55072) | 89 kDa | 32 | 41% |
|  |  | amplaxin [Homo sapiens] | [Q14247](http://www.uniprot.org/uniprot/Q14247) | 62 kDa | 20 | 38% |
|  |  | 26S proteasome subunit p97 [Homo sapiens] | [Q13200](http://www.uniprot.org/uniprot/Q13200) | 100 kDa | 16 | 20% |
|  |  | Ubiquitin-like modifier activating enzyme 2 [Homo sapiens] | [Q9UBT2](http://www.uniprot.org/uniprot/Q9UBT2) | 71 kDa | 15 | 29% |
|  |  | Striatin, calmodulin binding protein 3 [Homo sapiens] | [A0AV58](http://www.uniprot.org/uniprot/A0AV58) | 78 kDa | 12 | 29% |
|  |  | elongation factor-2 kinase [Homo sapiens] | [O00418](http://www.uniprot.org/uniprot/O00418) | 82 kDa | 4 | 5% |
|  |  | signaling adaptor protein DIP13alpha [Homo sapiens] | [Q9UKG1](http://www.uniprot.org/uniprot/Q9UKG1) | 80 kDa | 3 | 6% |
|  |  | ACTG1 protein [Homo sapiens] | [P63261](http://www.uniprot.org/uniprot/P63261) | 42 kDa | 2 | 7% |
| **1,45** | **1165** | G patch domain and KOW motifs [Homo sapiens] | [Q92917](http://www.uniprot.org/uniprot/Q92917) | 52 kDa | 15 | 33% |
|  |  | P58 [Homo sapiens] | [P30101](http://www.uniprot.org/uniprot/P30101) | 57 kDa | 9 | 24% |
|  |  | UDP-glucose dehydrogenase [Homo sapiens] | [O60701](http://www.uniprot.org/uniprot/O60701) | 55 kDa | 3 | 9% |
|  |  | ALB protein [Bos taurus] | [P02769](http://www.uniprot.org/uniprot/P02769) | 69 kDa | 2 | 8% |
|  |  | FBLIM1 protein [Homo sapiens] | [Q8WUP2](http://www.uniprot.org/uniprot/Q8WUP2) | 41 kDa | 2 | 7% |
|  |  | translation initiation factor 5 [Homo sapiens] | [P55010](http://www.uniprot.org/uniprot/P55010) | 49 kDa | 2 | 4% |
|  |  | Misato homolog 1 (Drosophila) [Homo sapiens] | [Q9BUK6](http://www.uniprot.org/uniprot/Q9BUK6) | 62 kDa | 2 | 5% |
|  |  | Adenylyl cyclase-associated protein 2 [Cricetulus griseus] | G3GUF9 | 50 kDa | 2 | 6% |
| **1,27** | **747** | metalloproteinase [Homo sapiens] | [P52888](http://www.uniprot.org/uniprot/P52888) | 79 kDa | 13 | 21% |
|  |  | CTP synthase [Homo sapiens] | [P17812](http://www.uniprot.org/uniprot/P17812) | 67 kDa | 13 | 19% |
|  |  | Transferrin [Bos taurus] | [Q29443](http://www.uniprot.org/uniprot/Q29443) | 78 kDa | 9 | 14% |
|  |  | neurolysin [Homo sapiens] | [Q9BYT8](http://www.uniprot.org/uniprot/Q9BYT8) | 81 kDa | 8 | 11% |
|  |  | ALB protein [Bos taurus] | [P02769](http://www.uniprot.org/uniprot/P02769) | 69 kDa | 3 | 4% |
|  |  | general transcription factor IIF subunit 1 [Homo sapiens] | [P35269](http://www.uniprot.org/uniprot/P35269) | 58 kDa | 3 | 11% |
| **1,26** | **1030** | Phosphoglucomutase 1 [Homo sapiens] | [P36871](http://www.uniprot.org/uniprot/P36871) | 61 kDa | 13 | 25% |
|  |  | arginine-tRNA-protein transferase 1-2p [Homo sapiens] | [O95260](http://www.uniprot.org/uniprot/O95260) | 58 kDa | 4 | 13% |
|  |  | heterogeneous nuclear ribonucleoprotein L [Homo sapiens] | [P14866](http://www.uniprot.org/uniprot/P14866) | 64 kDa | 3 | 6% |
|  |  | Receptor-interacting serine-threonine kinase 2 [Homo sapiens] | [O43353](http://www.uniprot.org/uniprot/O43353) | 61 kDa | 2 | 4% |
| **1,36** | **1203** | UDP-glucose dehydrogenase [Homo sapiens] | [O60701](http://www.uniprot.org/uniprot/O60701) | 55 kDa | 9 | 19% |
|  |  | UBX domain-containing protein 1 [Homo sapiens] | [Q9BZV1](http://www.uniprot.org/uniprot/Q9BZV1) | 50 kDa | 4 | 15% |
|  |  | CAP protein [Homo sapiens] | [D3DPU2](http://www.uniprot.org/uniprot/D3DPU2) | 52 kDa | 3 | 7% |
|  |  | Chromosome 22 open reading frame 28 [Homo sapiens] | [Q9Y3I0](http://www.uniprot.org/uniprot/Q9Y3I0) | 55 kDa | 2 | 4% |
|  |  | glucose-6-phosphate 1-dehydrogenase isoform b [Homo sapiens] | [P11413](http://www.uniprot.org/uniprot/P11413) | 59 kDa | 2 | 5% |
|  |  | aldehyde dehydrogenase [Homo sapiens] | [P00352](http://www.uniprot.org/uniprot/P00352) | 55 kDa | 2 | 6% |
|  |  | IMP (inosine monophosphate) dehydrogenase 2 [Homo sapiens] | [P12268](http://www.uniprot.org/uniprot/P12268) | 56 kDa | 2 | 5% |
| **-1,28** | **1844** | Aldo-keto reductase family 1, member B10 (aldose reductase) [Homo sapiens] | [O60218](http://www.uniprot.org/uniprot/O60218) | 36 kDa | 6 | 27% |
|  |  | glyceraldehyde-3-phosphate dehydrogenase [Homo sapiens] | [P04406](http://www.uniprot.org/uniprot/P04406) | 36 kDa | 3 | 12% |
|  |  | fructosamine-3-kinase [Homo sapiens] | [Q9H479](http://www.uniprot.org/uniprot/Q9H479) | 35 kDa | 3 | 13% |
| **-1,11** | **796** | Protein disulfide isomerase family A, member 4 [Homo sapiens] | [P13667](http://www.uniprot.org/uniprot/P13667) | 73 kDa | 10 | 16% |
|  |  | 2-oxoglutarate and iron-dependent oxygenase domain containing 1 [Homo sapiens] | [Q8N543](http://www.uniprot.org/uniprot/Q8N543) | 63 kDa | 8 | 18% |
|  |  | Sorting nexin 1 [Homo sapiens] | [Q13596](http://www.uniprot.org/uniprot/Q13596) | 59 kDa | 8 | 17% |
|  |  | BiP [Homo sapiens] | [P11021](http://www.uniprot.org/uniprot/P11021) | 72 kDa | 3 | 7% |
|  |  | Sorting nexin 2 [Homo sapiens] | [O60749](http://www.uniprot.org/uniprot/O60749) | 58 kDa | 3 | 9% |
|  |  | golgi resident protein GCP60 [Homo sapiens] | [Q9H3P7](http://www.uniprot.org/uniprot/Q9H3P7) | 61 kDa | 3 | 10% |
| **-1,67** | **1033** | T-complex 1 [Homo sapiens] | [P17987](http://www.uniprot.org/uniprot/P17987) | 60 kDa | 29 | 46% |
|  |  | Coiled-coil domain containing 6 [Homo sapiens] | [Q16204](http://www.uniprot.org/uniprot/Q16204) | 53 kDa | 5 | 13% |
|  |  | ALB protein [Bos taurus] | [P02769](http://www.uniprot.org/uniprot/P02769) | 69 kDa | 3 | 6% |
|  |  | hCRMP-2 [Homo sapiens] | [Q16555](http://www.uniprot.org/uniprot/Q16555) | 62 kDa | 3 | 9% |
|  |  | zinc finger protein 622 [Homo sapiens] | [Q969S3](http://www.uniprot.org/uniprot/Q969S3) | 54 kDa | 2 | 5% |
|  |  | Kinesin light chain 1 [Homo sapiens] | [Q07866](http://www.uniprot.org/uniprot/Q07866) | 64 kDa | 2 | 5% |
| **-1,21** | **1213** | CAP protein [Homo sapiens] | [D3DPU2](http://www.uniprot.org/uniprot/D3DPU2) | 52 kDa | 22 | 52% |
| **-1,17** | **2009** | EB1 [Homo sapiens] | [Q15691](http://www.uniprot.org/uniprot/Q15691) | 30 kDa | 7 | 41% |
|  |  | ribosomal protein S2 [Mus musculus] | [Q58EU3](http://www.uniprot.org/uniprot/Q58EU3) | 31 kDa | 3 | 12% |
