# Supplementary material for: miRNA-196b inhibits cell proliferation and induces apoptosis in HepG2 cells by targeting IGF2BP1
Source: Mol Cancer. 2015 Apr 8;14:79. doi: 10.1186/s12943-015-0349-6 (PMC4403945; doi:10.1186/s12943-015-0349-6)
Supplement: Additional file 2: — Supplementary methods. [file 12943_2015_349_MOESM2_ESM.docx]

**Additional file 2:** Supplementary methods

***Sample preparation for 2-D DIGE analysis***

Protein concentrations were determined with Pierce 660 assay (Thermo Scientific, Waltham, MA, USA) and adjusted at 5 μg/μl. Four independent replicates were used for each experimental condition. The pH of the protein extract was adjusted to 8.5 by addition of the appropriate volume of 50 mM NaOH. The protein labeling with the cyanine dyes (GE Healthcare, Uppsala, Sweden) was performed according to the manufacturer’s instructions with minimal dye labeling. Cy3 and Cy5 were used to label pre-miR negative control and pre-miR-196b samples, while Cy2 was used to label a pooled standard. Briefly, 25 μg of proteins from each sample to be compared (pre-miR CTL- and pre-miR-196b samples) were labeled with 200 pmol of dye (Cy3 or Cy5). To eliminate dye specific differences, samples from pre-miR negative control or pre-miR-196b were labeled cross-wise with Cy3 and Cy5 for one of each four replicates. A pooled standard of 25 μg composed of equal amount of proteins from the 8 experimental conditions (pre-miR negative control and pre-miR-196b in four replicates) was labeled with 200 pmol of Cy2 that represented the internal standard on each gel. After 30 min on ice in the dark, the labeling was stopped by the addition of 10 mM lysine for 10 min.

***Two-Dimensional In-Gel Electrophoresis***

An amount of 25 μg of each individual labeled sample (pre-miR CTL-, pre-miR-196b and pooled standard samples from four replicate experiments) were combined. The three labeled mixtures were combined and the total proteins (75 μg) were added v:v to reducing buffer (7 M urea, 2 M thiourea, 20 mM DTT, 4 % CHAPS, 2 %, IPG 3–11 buffer) for 15 min at room temperature, in the dark and then incubated at room temperature for 10 min to allow a better reduction before centrifugation for 5 min at 13 000 rpm.

Immobilized pH gradient strips (18 cm, pH 3-11; GE Healthcare) were passively rehydrated overnight with 450 μl of a rehydration solution (7 M urea, 2 M thiourea, 2 % CHAPS, 0.5 % IPG 3-11 buffer, 2 % DTT). Sample sets containing the labeled mixtures were cup-loaded onto the IPG strips and isoelectric focusing was performed with an EttanTM IPGphor II isoelectric focusing unit (GE Healthcare). IEF conditions were as follows : 300 V for 3h, a gradient of 1 000 V for 8h, followed by a gradient of 8 000 V for 3h and then 25 000 V / h (20°C) and a maximum current setting of 50 μA per strip. Prior to second-dimension separation, each IPG strip was then reduced (10 mg/mL DTT) for 15 min and alkalized (25 mg/mL iodoacetamide) for 15 min in equilibration buffer (1.5 M Tris-HCl pH 8.8, 6 M urea, 30 % glycerol and 2 % SDS) just before loading onto a 12.5 %, 18 cm, 1 mm thick acrylamide gel. The strips were overlaid with 1% agarose in SDS running buffer (25 mM Tris, 192 mM glycine, 0.1 % SDS) and migrated in electrophoresis 0.5 W per gel for 1 h, 1.5 W per gel for 16 h and 1 W for 2 h on a EttanDALT II system (GE Healthcare).

***Gel scanning and image analysis***

Labeled proteins separated in the 2D gel were visualized using a Typhoon 9400 imager (GE Healthcare) at appropriate wavelength to detect the three cyanine dyes. Gels were scanned at wavelengths specific for the CyDyes (488 nm for Cy2, 532 nm for Cy3, and 633 nm for Cy5).

The resolution was 100 μm. Determination of protein spot abundance and statistical analyses were carried out automatically using the DeCyder 2D Differential Analysis software version 6.0 (GE Healthcare). The four CyDye-labeled forms of each spot were co-detected within each gel. Ratios between sample (pre-miR CTL- versus pre-miR-196b) and internal standard abundances were calculated for each protein spot with the DIA (Differential In-gel Analysis) module. Inter-gel variability was corrected by matching of the internal standard spot maps by the BVA (Biological Variance Analysis) module of the DeCyder software that allows statistical analysis of protein abundance changes in the different gels. Protein spots with statistically significant Student’s *t*-test (p < 0.05) and more than 1.2-fold change in volume after normalization (between the two types of samples in the four replicates) were considered as differentially intense.

***Spot excision and protein digestion***

Spots were excised from gels using the Ettan™Spot Picker (GE Healthcare) and proteins were digested with trypsin by in-gel digestion. The gel pieces were washed twice with distilled water and then shrunk with 100 % acetonitrile. The proteolytic digestion was performed by the addition of 3 μl of modified trypsin Gold Mass (12.5 ng/µL, Promega) in 100 mM NH_4_HCO_3_ cold buffer. Proteolysis was performed overnight at 37 °C. The supernatant was collected and kept at −20 °C prior to analysis.

***Protein identification and mass spectrometry***

Peptides were analyzed by using nano-LC-ESI-MS/MS maXis Impact UHR-TOF (Bruker, Bremen, Germany) coupled with a Thermo Fischer UltiMate 3000 (Thermo Fischer, Waltham, MA, USA). The digests were separated by reverse-phase liquid chromatography using a 75 µm x 150 mm reverse phase Thermo Fischer (Acclaim PepMap 100 C18). MaXis and Ultimate systems were piloted by Compass HyStar 3.2 (Bruker). Peak lists were created using DataAnalysis 4.1 (Bruker) and saved as XML file for use with ProteinScape 3.0 (Bruker) with Mascot 2.3 as search engine (Matrix Science). Enzyme specificity was set to trypsin, and the maximum number of missed cleavages per peptide was set at one. Carbamidomethylation was allowed as fixed modification and oxidation of methionine Gln to pyro-Glu as variable modifications. The peak lists were searched against the mammalian taxonomy of the National Center for Biotechnology Information non redundant (NCBInr) database (2075986 sequences). Scaffold (version Scaffold 4.3.4, Proteome Software Inc., Portland, OR, USA) was used to validate MS/MS based peptide and protein identifications. All MS/MS samples were analyzed using Mascot (Matrix Science, London, UK; version 2.3) and X!Tandem (The GPM, thegpm.org; version 2010.12.01). Peptide identifications were accepted if they could be established with a probability greater than 95 %. Protein identifications were accepted if they could be established with a probability greater than 99 % and at least 2 identified peptides, as specified by the Peptide Prophet algorithm [[1](#_ENREF_1)]. Protein probabilities were assigned by the Protein Prophet algorithm [[2](#_ENREF_2)].

1. Keller A, Nesvizhskii AI, Kolker E, Aebersold R: **Empirical statistical model to estimate the accuracy of peptide identifications made by MS/MS and database search.** *Anal Chem* 2002, **74:**5383-5392.

2. Nesvizhskii AI, Keller A, Kolker E, Aebersold R: **A statistical model for identifying proteins by tandem mass spectrometry.** *Anal Chem* 2003, **75:**4646-4658.
